# Agricultural practices and pollinators modulate the anthosphere microbiome

**DOI:** 10.1093/ismeco/ycaf026

**Published:** 2025-02-12

**Authors:** Jari Temmermans, Marie Legein, Ilaria Checchia, Giovanna E Felis, Wenke Smets, Reet Karise, Sarah Lebeer

**Affiliations:** Laboratory of Applied Microbiology & Biotechnology, Department of Bioscience Engineering, Antwerp University, Groenenborgerlaan 171, Antwerp 2020, Belgium; Laboratory of Applied Microbiology & Biotechnology, Department of Bioscience Engineering, Antwerp University, Groenenborgerlaan 171, Antwerp 2020, Belgium; Department of Biotechnology, University of Verona, Villa Lebrecht, Via della Pieve 70, San Pietro in Cariano 37029, Italy; Department of Biotechnology, University of Verona, Villa Lebrecht, Via della Pieve 70, San Pietro in Cariano 37029, Italy; VUCC-DBT, Department of Biotechnology, Verona University Culture Collection, University of Verona, Strada Le Grazie 15, Ca' Vignal 2, Verona, VR 37134, Italy; Laboratory of Applied Microbiology & Biotechnology, Department of Bioscience Engineering, Antwerp University, Groenenborgerlaan 171, Antwerp 2020, Belgium; Chair of Plant Health, Institute of Agricultural and Environmental Sciences, Estonian University of Life Sciences, Kreutzwaldi 1, Tartu 51006, Estonia; Laboratory of Applied Microbiology & Biotechnology, Department of Bioscience Engineering, Antwerp University, Groenenborgerlaan 171, Antwerp 2020, Belgium

**Keywords:** anthosphere, microbiome, cultivation intensification, pollinator, strawberry, flower

## Abstract

The flower microbiome is pivotal in plant health, influencing reproductive success, fruit quality, and pathogen vulnerability. However, the impact of intensified agricultural practices on these microbial communities remains to be understood. This study examines how specific agricultural practices influence the bacterial composition of the strawberry anthosphere, focusing on cultivation intensification. Intensified systems were defined by practices such as indoor glasshouse substrate-based cultivation, increased use of plant protection products, larger cultivation areas, and reliance on managed pollinators. Using citizen science and V4 16S rRNA gene sequencing, we found that flowers in these more intensively managed systems had lower bacterial diversity, more variable microbiomes, and loss of core taxa such as *Sphingomonas* and *Pseudomonas*. To determine if pollinators could help mitigate these effects, we conducted exclusion experiments. In a tunnel system, we observed that foraging pollinators facilitated the dispersal of specific bacteria, such as *Staphylococcus* and *Pseudomonas*, and increased flower bacterial richness. However, in an open field, foraging pollinators had no significant impact. Our findings highlight the significant impact of cultivation intensification on the anthosphere microbiome and suggest that pollinators may play a role in restoring microbiome diversity. This research fills a critical gap in understanding how agricultural practices shape plant microbiomes and underscores the potential for microbe-based strategies to improve plant health in intensively managed systems.

## Graphical Abstract

**Graphical Abstract:**
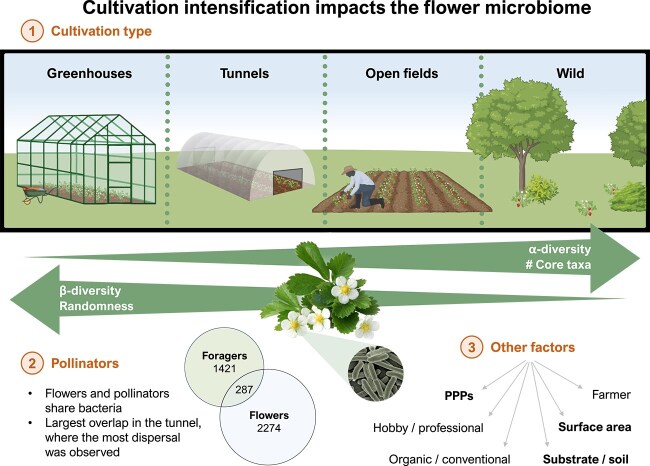

## Introduction

Microbial communities inhabit every plant surface and their contribution to plant growth [1], pathogen resilience [2–4], stress resilience [5, 6] and even fruit taste and aroma [7–9] has been well established. While agricultural practices aim to optimize these plant properties, they can also inadvertently disrupt plant-associated microbial communities, potentially compromising these benefits. Most research on the impact of agriculture has focused on soil and rhizosphere microbes (microbes associated with below-ground parts of the plant), which appear to be affected by tillage [10], organic versus conventional management type [10, 11], and land-use intensity [12, 13]. In addition, the process of plant domestication has been shown to affect the root microbiome in several crops [14–16], potentially leading to a loss of interactions with microbial commensals and a more profound reliance on plant protection products compared to their wild counterparts [15, 17].

Studies on the phyllosphere microbiome (microbes associated with above-ground plant parts, primarily leaves) have shown that pesticides can induce a shift in the leaf microbial community composition [18, 19]. Moreover, when plants are moved out of the soil and cultivated in greenhouses or indoor systems, phyllosphere communities have shown reduced diversity and abundance due to limited bacterial dispersal sources such as soil, insects, and surrounding vegetation [20–22]. Consequently, horticultural crops and berries are increasingly losing microbial symbionts as their cultivation shifts toward more intensive practices, including tunnels, greenhouses, and vertical indoor cultivation systems [23–25]. Simultaneously, these crops often rely heavily on fungicides, with strawberries ranking at the top of the “Dirty Dozen” list since at least 2016 (https://www.ewg.org/foodnews/dirty-dozen.php).

Though less studied, the anthosphere microbiome (microbes associated with the flowers) is vital for plant reproduction [26, 27], fruit quality [7, 8], and resistance against pathogens such as *Botrytis cinerea* and *Erwinia* [26, 28, 29]. Like the phyllosphere, the anthosphere is influenced by the surrounding environment and atmosphere, but it is unique in several ways. It is ephemeral, giving microbial communities only a short window to develop [30], it contains high concentrations of sugars and proteins in nectar and pollen [26], and thirdly, it is visited by pollinators, which can act as vectors for microbial dispersal [31]. Therefore, understanding the impact of agricultural practices on the anthosphere microbiome is highly relevant for agricultural applications.

To investigate these effects involving all stakeholders, we conducted a citizen-science study across Belgium, The Netherlands, Germany and Estonia. By involving local strawberry growers, the citizen-science approach enabled broad data collection across diverse environments, offering insights into how commercial practices impact the flower microbiome at a regional scale. Growers and researchers sampled strawberry flowers in the wild, open fields, tunnels and greenhouses and reported on their cultivation practices through a questionnaire. We analysed how the flower microbiome varied across a gradient of cultivation intensification. Additionally, we conducted pollinator exclusion experiments in a tunnel and open-field system to assess the role of pollinators in anthosphere microbiome development. We hypothesized that pollinators introduce microbes and therefore affect the flower microbial community, potentially aiding the development of a natural-resembling flower microbiome, especially within microbe-poor cultivation systems.

## Materials and methods

### Sampling of strawberry flower microbiome

To involve a variety of commercial strawberry growers with open fields, tunnels and greenhouses, we initiated a cocreational citizen-science project named Sabofleur. This project, launched on August 27, 2022, aimed to engage growers in collaborative research to study the impact of cultivation practices on the anthosphere microbiome. Strawberry growers registered by responding to three online questions regarding the cultivation type (greenhouses vs tunnels vs open fields), expected flowering period in 2023 and address to receive a Sabofleur sampling kit. Each Sabofleur Kit, provided to growers, included three empty 50 ml tubes for the collection of one strawberry flower, ethanol wipes, a pair of nitrile gloves, a paper questionnaire that asked participants about their cultivation practices, and a comprehensive brochure featuring infographics and instructions in layman’s terms to ensure consistency of sampling (Supplementary Fig. 1). Following the growers’ sampling efforts, the tubes were placed inside a cardboard box and transported at ambient temperature with prepaid services by the national parcel service (Bpost for Belgium or PostNL for The Netherlands), a familiar service for all participants. Upon arrival, 3 ml of eNAT buffer (Copan) was added to each 50 ml tube. No buffer was added during transport to ensure the safety of all participating growers. To estimate and account for the effect of transport, we recorded the transport time of all samples and included this as a variable in the PERMANOVA analysis. In total 39 greenhouse samples, 21 tunnel samples, and 30 open-field samples arrived via the parcel service. Additionally, 36 wildflowers from 10 locations were collected and transported to the lab on the same day. In the lab, all sample tubes were vortexed and subsequently stored at −20°C until further processing.

**Figure 1:**
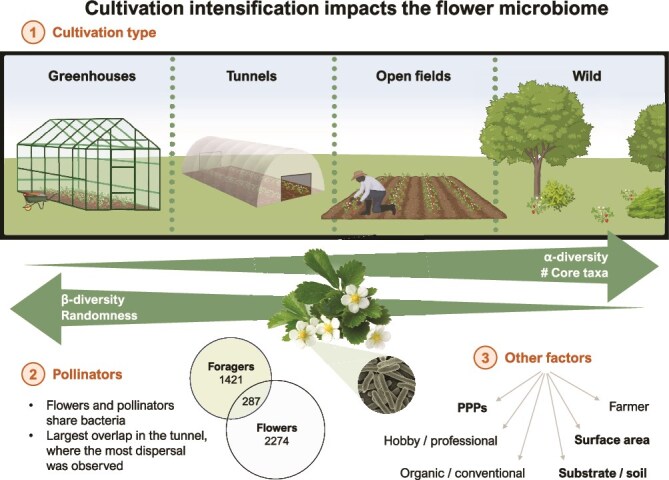
Graphical abstract: Effect of cultivation type, pollinators, plant protection products, substrate type and other factors on the strawberry anthosphere bacterial community.

### Microbiome sequencing and analysis

After storage, each sample was vortexed for 5 min to resuspend the bacteria. Next, 500 μl of eNAT per tube was extracted with the PowerFecal DNA isolation kit (Qiagen, Hilden, Germany) with automated processing on the QIAcube (Qiagen), with an elution volume of 80 μl, according to the manufacturer’s instructions. Two blanks per extraction kit were included: one in the first well and one in the last of each 96-well plate. Content and read counts of the blanks can be found in Supplementary Fig. 9. No specific contaminants were removed, but a random non-consistent amount of cross-contamination was observed, as is often the case, especially using a DNA Extraction robot [32]. The extracted DNA served as the template for amplifying the V4 region (same as the earth microbiome project, https://earthmicrobiome.org/protocols-and-standards/16s/) of the 16S rRNA gene using Phusion High-Fidelity DNA polymerase (Thermo Fisher Scientific Waltham, Massachusetts, USA). We used standard barcoded forward and reverse primers, following the protocol outlined by Kozich et al. (2023) [33]. In each 20 μl reaction, 7 μl of extracted DNA was added. Additionally, two PCR blanks were included per 96-well plate and sequenced alongside the samples. The thermocycler (Eppendorf AG, Mastercycler nexus gradient) was programmed with the following parameters: an initial denaturation step at 95°C for 2 min, followed by 30 cycles at 95°C for 20 s, 75°C for 10 s, 55°C for 20 s, 72°C for 1 min, and a final extension at 72°C for 10 min.

Next, the amplicons were checked on a 1% (mass/vol) agarose gel and purified using the Agencourt AMPure XP Magnetic Bead Capture Kit (Beckman Coulter, Bria, California, USA). Based on the band brightness on agarose gel, samples and blanks were pooled in near equimolar concentrations, resulting in a library. The amplicon library was further purified by running it on a 0.8% (mass/vol) agarose gel and extracting bands of ~380 bp with the Nucleospin Gel and PCR Clean-up kit (Macherey-Nagel, Düren, Germany). The final library was diluted to 2 nM using the Qubit 3.0 Fluorometer (Life Technologies, Carlsbad, California, USA) and sequenced on the Illumina MiSeq platform using 2 × 250 cycles at the Center of Medical Genetics Antwerp (University of Antwerp, Belgium).

Quality control and processing of amplicon reads were performed with the R package DADA2 [34]. Briefly, reads with over two expected errors were removed. Forward and reverse reads were denoised per sample using the DADA2 algorithm and subsequently merged. Chimeras were removed using the removeBimeraDenovo function. The merged and denoised reads (amplicon sequence variants, ASVs) were taxonomically annotated from the phylum to the genus level with the assignTaxonomy function using the GTDB (release R207) reference 16S rRNA database [35]. Nonbacterial reads (i.e. plastid and mitochondrial DNA) and ASVs with a length greater than 260 bases were removed from the data set. Additionally, samples containing fewer than 500 reads were discarded. Further data analysis was done in the R environment, using the tidyverse package [36] and an in-house built package, tidytacos (https://github.com/LebeerLab/tidytacos).

### Open field pollinator experiment

The open field was an organically maintained strawberry field near Tartu, Estonia, where plants of the cultivars Asia (most), Sonata, and Honeoye were cultivated. To exclude large pollinators, two designated zones containing 5–10 strawberry plants, were netted one week before the experiment on 23/05/2023, and open flowers were carefully removed. This approach allowed all open flowers sampled during the experiment to bud while under the net. After 6 days (29/05), flowers beneath the nets were manually pollinated using sterile swabs. Approximately 100 m from the strawberry field, 12 honeybee (*Apis mellifera*) hives were kept. Honeybees could forage ad libitum on the strawberry field except for the netted plants and the surrounding rapeseed oil fields. One week later, we sampled 25 flowers from the netted plants, 24 flowers from plants not covered by a net, 10 honeybees foraging in the strawberry field, 11 honeybees near hive entrance while opening the hives, 20 nursing bees collected from the brood frame and 20 honeybee pupae from a total of four different hives. Adult honeybees and flowers were stored in 50 ml tubes while pupae were preserved in 2 ml Eppendorfs. After transportation to the lab on the same day at ambient temperature, eNAT buffer was added, 3 ml for flowers and adult honeybees and 1 ml for pupae. All samples were vortexed and subsequently stored at −20°C until further processing. One month later, samples were transported on dry ice to Belgium and stored again at −20°C on 05/07/2023 for subsequent analysis, analogously to above. Pollinator samples that did not show any bands after gel electrophoresis (17 pupae, three open field foragers, and one honeybee outside the hive) were left out.

### Tunnel field pollinator experiment

The tunnel experiment was conducted at the Research Centre Hoogstraten (Proefcentrum Hoogstraten, Hoogstraten, Belgium). Before flowering, nets were installed on 14 strawberry plants in various zones of the same row in a semi-closed tunnel (29/06/2023). After 5 days, (04/07/2023), 49 flowers (21 from beneath the covers and 28 from outside the covers) and 20 pollinators (17 wild pollinators and three commercially reared bumblebees, Biobest, Westerlo, Belgium) were sampled and transported to the lab on the same day at ambient temperature. Analogously to the open field experiment, all samples were preserved in 50 ml tubes, vortexed and stored at −20°C in 3 ml eNAT buffer.

To quantify the shared bacteria between flowers and pollinators within each environment, we adapted the transfer index method described by Legein et al. (2022) [20] to account for highly abundant taxa. Consequently, the transfer of bacteria was quantified as the proportion of unique flower ASVs that also occur in forager samples within the same environment, relative to the total amount of ASVs on the flower, with a minimum absolute abundance larger than three reads.

### Molecular identification of pollinators

DNA was extracted from the insect specimens’ thorax, using the DNeasy Blood & Tissue kit (Qiagen, Milan, Italy) following the manufacturer’s instructions. The genomic DNA samples were stored at −20°C for subsequent analysis.

Afterwards, target DNA from mitochondrial gene Cytochrome Oxidase subunit I (COI) was amplified using the forward primer LCO1490 (5′- GGTCAACAAATCATAAAGATATTGG-3′) and reverse primer HCO2198 (5′- TAAACTTCAGGGTGACCAAAAAATCA-3′) [37]. The PCR program consisted of 30 s of denaturation at 94°C, fivecycles of 10 s at 94°C, 20 s at 45°C, 25 s at 72°C, 35 cycles of 10 s at 94°C, 20 s at 45°C, 25 s at 72°C, and 1 min at 72°C, then 2 min at 72°C for the final elongation. The reagent concentrations were as follows: DreamTaq Green Buffer (Thermo Scientific) 1X, dNTPs 200 μM, primer LCO1490 0.4 μM, primer HCO2198 0.4 μM, Dream Taq (Thermo Scientific) 1.25 U and DNA 20 ng/μl.

Next, PCR products were analysed on a 1.0% (mass/vol) agarose gel for 40 min at 110 V in 1× TAE buffer (40 mM Tris, 20 mM acetic acid, and 0.4 mM EDTA) stained with EuroSafe colorant Acid Stain. Visualization and image capture were performed under UV with a ChemiDocXRS+ Imaging System (Bio-Rad, Segrate, Italy). The size of the DNA fragments (700 bp) was compared with the GeneRuler 1 kb DNA Ladder (Thermo Scientific) by agarose gel electrophoresis.

Following gel electrophoresis, PCR products were purified using the NucleoSpin® Gel and PCR Clean-up kit (CARLO ERBA, Milan, Italy) and subsequently sent for sequencing at Eurofins Genomics (Ebersberg, Germany). Sequencing data were analysed by comparing with the GenBank database using the BLAST alignment tool (http://blast.ncbi.nlm.nih.gov/, accessed on 7 February 2024).

### Statistical analysis

All statistical analyses were performed with R 4.2.2. To evaluate how bacterial alpha diversity responded to agricultural practices, we used linear mixed models. Each mixed-effect model included the read count per sample as a random effect. Alpha diversity was log-transformed.

The bacterial beta diversity (Bray–Curtis dissimilarity based on ASV relative abundances within samples) was evaluated using permutational multivariate analyses of variance models (PERMANOVA, adonis2 function, 999 permutations) to identify factors explaining variation in community composition. As the order of the variables determines their importance, with the first factors absorbing variation from subsequent factors, the most general sample characteristic, i.e. farmer, was entered last and the more specific characteristics i.e. cultivation type, first.

Moreover, the betadisper function from the vegan R package [38], followed by ANOVA was used to detect changes in the community dispersion based on the Bray-Curtis dissimilarities, calculated using the vegdist function from the vegan package. The relationship between the Bray–Curtis dissimilarity of bacterial communities and the numerical variable cultivation area (m^2^) was examined, performing constrained analysis of principal coordinates (CAP), using the capscale function from the vegan package in R.

Core ASVs on flowers in each cultivation type were determined using the occupancy-abundance elbow approach, developed especially for plant-associated microbiomes by Shade et al. (2019) [39]. Neutral models were fitted to the occupancy-abundance curves using the R code from Burns et al. (2016) [40] to fit the neutral model from Sloan et al. (2007) [41]. Samples had to be rarefied to 500 ASVs to match the model for this analysis.

Microbiome differential abundance analysis (https://github.com/thiesgehrmann/multidiffabundance) was conducted to identify taxa exhibiting significant preferences for a specific cultivation type, utilizing algorithms including maaslin2 [42], lmclr, limma [43], deseq2 [44] and aldex2 [45].

## Results

### Anthosphere microbiome sequencing

In total, 27 growers participated in the Sabofleur citizen-science project, collectively sampling across 30 locations between March and August 2023. Samples arrived in the lab with an average transport time of 3.57 ± 4.55 days (n = 30) with 86.7% arriving within 7 days from sampling.

After quality control, Illumina sequencing, targeting the V4 region of the 16S rRNA gene region, yielded a total of 3.4 million read pairs, with an average of 33 375 high-quality reads per sample across 102 flower samples encompassing greenhouses (n = 19/39), tunnels (n = 17/21), open fields (n = 30/30), and the wild (n = 36/36). Wildflower samples were collected from two different species, *Fragaria vesca* (n = 24) and *Potentilla indica* (n = 12), while commercial flowers were sampled from *Fragaria* × *ananassa* plants. After quality filtering and removing eukaryote-originating sequences, 6932 distinct ASVs were identified. The abundances of the 10 most abundant taxa in flower samples are visualized per sample in Supplementary Fig. 2.

### Cultivation practices impact the flower microbiome

PERMANOVA revealed that the bacterial community composition was most strongly associated with the cultivation type: greenhouses vs open fields vs tunnels (R^2^ = 0.11), followed by other factors shown in Table 1. Furthermore, sheltered cultivation was associated with applying plant protection products (PPPs), as they were used in eight out of nine greenhouses, while only in four out of seven tunnel systems and five out of 10 open fields. Moreover, in the greenhouse, all cultivation was substrate-based whereas only two out of seven tunnels and two out of 10 open fields used a substrate instead of soil. The effects of specific factors were further investigated.

**Table 1:** Permutational multivariate analyses of variance (PERMANOVA) of all factors included in this study (number of permutations = 9999). Factors are in the order they were added to the model. ^*^P < .05, ^**^P < .01, ^***^P < .001.

Factor	R^2^	p-value	df	F-value
Cultivation type	0.11	0.0001	2	5.77
Cultivated surface area	0.03	0.0006	1	3.16
Soil/substrate	0.04	0.0001	1	4.63
(Un)treated	0.03	0.0012	1	2.97
Commercial/natural pollination	0.03	0.0006	1	3.04
Organic/conventional	0.02	0.025	1	1.90
(Un)heated	0.01	0.73	1	0.76
Days in transit	0.04	0.0002	1	3.77
Hobby/professional	0.04	0.0005	2	2.35
Farmer	0.28	0.0001	13	2.29

**Figure 2:**
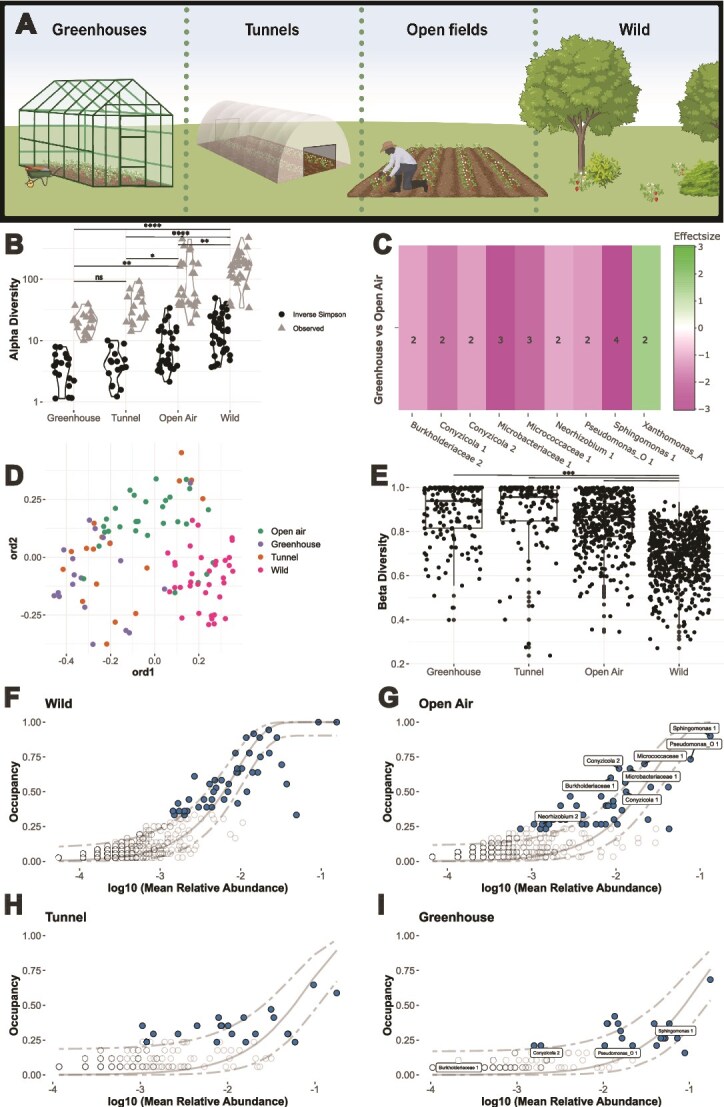
(A) Cultivation types sampled during the Sabofleur citizen science project (B) bacterial richness and inverse Simpson index in strawberry flowers per cultivation type with post-hoc Dunn p-value significance levels with Bonferroni correction for the inverse Simpson index. (C) MDA analysis via the Maaslin2 algorithm [42]: Greenhouse vs open-air environment. (D) PCOA plot (E) Beta diversity calculated among all samples within each cultivation type. P-value significance levels are the result of pairwise Betadisper analyses followed by ANOVA. All other pairwise comparisons were insignificant. (F–I) abundance-occupancy curves: The filled dots represent the ASVs that are part of the core community following the method by Shade et al (2019) [39] (Supplementary Fig. 10). Additionally, ASVs identified in (B) as significantly preferring open-field flowers over greenhouse flowers are labelled.

First, the cultivation type significantly impacted the flower bacterial diversity. Compared to open-air cultivation, bacterial richness was significantly different in greenhouses (*P =* .001), wildflowers (*P* = .001), and tunnels (*P* = .007) while accounting for the total number of reads per sample (*P* = .095). Both ASV richness and Simpson diversity followed an upward trend from most covered (greenhouses) to least (wildflowers) (Fig. 2B). Additionally, the estimated diversity by rarefaction curves followed the same upward trend from most covered to least (Supplementary Fig. 3).

Furthermore, Betadisper analysis followed by ANOVA revealed significant differences in the bacterial community dispersion of strawberry flowers among cultivation types (*P* = 1.28 × 10^−8^). Moreover, beta diversity, calculated among all flowers within the same cultivation environment, increased from least covered (wildflowers) to most (greenhouses) (Fig. 2E). A similar trend was observed when comparing beta diversity among flowers within the same commercial farm (Supplementary Fig. 4), showing that flowers within the same greenhouse were on average more dissimilar than flowers within the same open field.

Moreover, core ASVs (Supplementary information document), which are more likely to have a functional relationship with the plant host [39], were determined following Shade et al. (2019) [39]. A lack of a core community was observed in covered cultivation types, where highly abundant and prevalent ASVs were limited, conversely to the clear core community found in wildflowers (Fig. 2F–I). Notably, only one ASV (*Pseudomonas*_E1) occurred in over 50% of the samples in the greenhouse. Concurrently, the occupancy-abundance curve for wildflowers was a closer fit to the neutral community assembly model developed by Sloan et. al (2007) [41] (R^2^ = 0.76, RMSE = 0.06, Fig. 2F), suggesting a more natural balance between dispersal and selection in this ecosystem [39]. In contrast, cultivated flowers from open fields (R^2^ = 0.27, RMSE = 0.09, Fig. 2G), tunnels (R^2^ = −0.22, RMSE = 0.11, Fig. 2H) and greenhouses (R^2^ = 0.03, RMSE = 0.09, Fig. 2I) deviated more from the neutral model.

Microbiome differential abundance analysis was then conducted to identify taxa exhibiting significant preferences for a specific cultivation type. We compared the two extreme commercial environments: open fields and greenhouses. The same consistent results were obtained with each algorithm, described in the materials and methods section. The results of the maaslin2 algorithm (Fig. 2C) highlighted *Xanthomonas*_A as the only ASV occurring significantly more in greenhouses. While the short ASV sequence did not allow for species classification, the detected *Xanthomonas*_A ASV did not align with the well-known plant pathogen *Xanthomonas campestris*. Conversely, ASVs *Burkholderiaceae 1*, *Conyzicola* 1 and 2, *Microbacteriaceae* 1, *Micrococcaceae* 1, *Neorhizobium 2*, *Pseudomonas_O* 1, and *Sphingomonas* 1 occurred significantly more in open fields. Interestingly, all ASVs significantly more present in open-field flowers than greenhouse flowers were part of the open-field flower core microbiome (Supplementary Table 1).

We also compared the occupancy of all genera between cultivation types. Genera with a significantly higher prevalence (Fischer’s test) in one cultivation type over the other for each possible comparison were highlighted (Supplementary Fig. 5). Corresponding with the microbiome differential abundance analysis, *Xanthomonas* A emerged as the sole genus more prevalent in the greenhouse than in the open field. Additionally, other genera were identified as significantly more prevalent in open fields than greenhouses and tunnels. The most prevalent ones were *Sphingomonas*, *Sphingobium*, and *Massilia*. Supplementary Fig. 6 contains all taxa, aggregated on a genus level, which were significantly more prevalent in flowers grown in open fields than in greenhouses and tunnels.

Next, the effects on bacterial alpha diversity of applying plant protection products (PPPs), the second cultivation practice significantly influencing the flower bacterial community, were estimated. Specifically, when accounting for cultivation type and the total read count per sample (*P* = .07), introducing PPPs significantly (*P* = .03) reduced alpha diversity, quantified as the logarithm of the inverse Simpson index. Similarly, shifting from substrate-based to soil-based cultivation significantly reduced alpha diversity (*P* = .0009), after controlling for cultivation type and the total read count per sample (*P* = .2). The size of the cultivation area also significantly influenced flower bacterial alpha diversity, with effects varying based on cultivation type and whether the farming method was organic or conventional. In organic open fields, the inverse Simpson index showed a significant positive correlation with increasing surface area (*P* = .02), indicating greater microbial diversity in larger organic fields. However, this trend was reversed in covered cultivation environments, where a significant negative correlation (*P* = .003) between surface area and the inverse Simpson index was observed in tunnels, as analysed through a mixed linear interaction model. Furthermore, the positive correlation between surface area and the inverse Simpson index was significantly stronger in organic open fields than in conventional open fields (*P* = .03) (Supplementary Fig. 7A).

Similarly, CAP, followed by ANOVA revealed that the community composition variability within a field decreased in open fields of increasing size (Supplementary Fig. 7 B). In contrast, it increased in greenhouses and conventional tunnels of increasing size. Organic tunnels were an exception, as community composition in these tunnels remained more uniform.

### Pollinators transfer bacteria in open and sheltered cultivation environments

As shown by the PERMANOVA analysis (Table 1), introducing commercial pollinators appears to be significantly associated with the anthosphere community composition. Commercially reared bees were introduced in all nine greenhouses, in six of seven tunnels and three out of 10 open fields. Moreover, introducing commercial pollinators in tunnels was significantly associated with an increased inverse Simpson index as calculated in a general linear interaction model (*P* = .009), while accounting for the number of reads per sample (*P* = .29).

**Figure 3:**
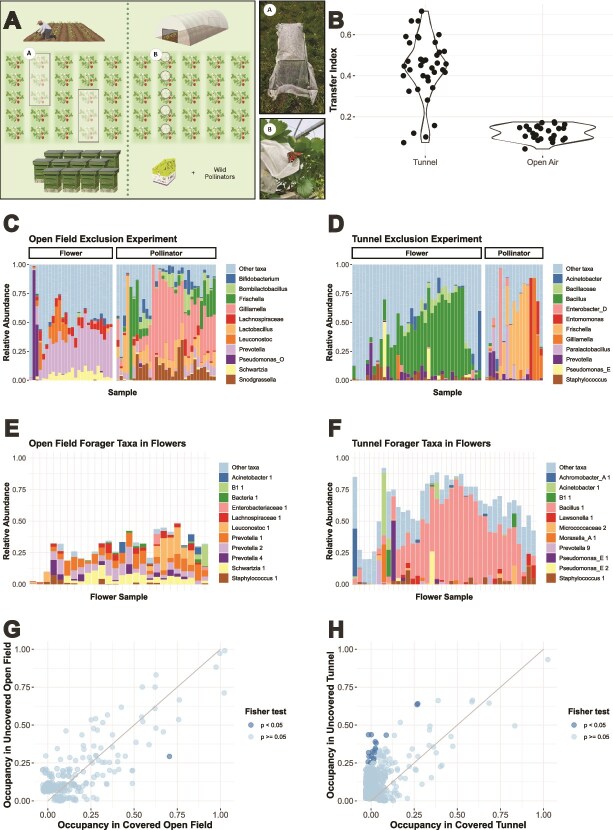
(A) Overview of both pollinator exclusion experiments. (B) Transfer index calculated as the proportion of unique ASVs in a flower (with a minimum absolute abundance >3 reads) also occurring in the forager samples in the same environment. (C) + (D) Relative abundance of bacterial genera on flowers and all pollinators in the open field and tunnel pollinator exclusion experiment. (E) + (F) Foraging pollinator taxa in covered and uncovered flowers on an ASV level in the tunnel and the open field. (G) + (H) Occupancy in netted vs unnetted strawberry flowers in an open field (G) and a commercially maintained strawberry tunnel (H). Genera that were significantly more prevalent in either netted or unnetted flowers were marked darker according to Fischer’s test.

To substantiate the active role of pollinators in introducing and dispersing bacteria in an agricultural environment, we performed an exclusion experiment in an open field and a tunnel system. Flower trusses were netted to exclude pollinators before flower opening (Fig. 3A). The open field was pollinated by reared honeybees (*A. mellifera*) and wild pollinators. The tunnel system was pollinated by reared bumblebees (*Bombus terrestris*) and wild pollinators. Of these, 18 specimens passed quality control and were used in the analysis. They were identified as reared *B. terrestris* [3], *Eristalis tenax* [10], *Bombus lapidarius* [1], *Eupeodes corollae* [2], and *Sphaerophoria philanthus* [1].

The open-field flowers harbored significantly more diverse bacterial communities than the tunnel flowers (inverse Simpson Wilcoxon *P* = 4.07x10^−5^). Moreover, no core taxa were observed in the tunnel except for one ASV, *Bacillus* 1, prevalent in 92% of flowers, and nine out of 17 pollinators. This ASV showed a 100% similarity over 253 base pairs with *Bacillus amyloliquefaciens* according to a blast in EZBioCloud, linking it to the weekly spraying of commercial product Taegro® (containing *B. amyloliquefaciens* FZB24) before, but not after, flower opening. Netting the flowers did not significantly affect the occupancy (Fischer *P* = .25) or relative abundance (Wilcox *P* = .21) of *Bacillus* 1.

Next, we identified taxa commonly associated with pollinators, flowers, or both. Figure 3 shows the 10 most abundant taxa in flowers and pollinators in the open field (panel 3C) and the tunnel (panel 3D). Panels 3E and 3F illustrate the 10 most abundant flower ASVs that were also found on foraging pollinators in both environments. In the open field, four of the shared ASVs belong to the genus *Prevotella*, with additional notable shared ASVs from the genera *Leuconostoc*, *Schwartzia, Staphylococcus*, and *Pseudomonas*. In the tunnel, shared ASVs included *Bacillus* 1 and, similarly to the open field, ASVs from the genera *Staphylococcus* and *Pseudomonas*. Subsequently, for both environments, we identified genera with significantly higher prevalence in either flowers or pollinators, based on Fischer’s test (Supplementary Fig. 8A and B). A list of all genera, significantly more prevalent in flowers or foraging pollinators, is presented in Supplementary Fig. 8C and D.

Last, we examined the impact of excluding pollinators on the community composition, bacterial dispersal on flowers, and richness. To assess bacterial dispersal by pollinators, we calculated the transfer index as the proportion of distinct pollinator-associated ASVs to the total distinct ASV count per flower in both the open field and tunnel system. Flowers contained a minimum of pollinator-associated ASVs with a minimal transfer index of 4.3% in the open field and 7.4% in the tunnel. However, the average transfer index was significantly higher in the tunnel, as 43.9% of distinct flower ASVs were also found on foraging pollinators, compared to an average of 12.3% in the open field (Wilcox *P* = .00017) (Fig. 3B).

Surprisingly, netting did not reduce the transfer proportion in either the open field or tunnel, and excluding pollinators did not alter the community composition in the open field (*P* = .88, R^2^ = 0.028) and only marginally in the tunnel (*P* = .08, R^2^ = 0.043) according to PERMANOVA. However, netting did decrease alpha diversity (inverse Simpson and observe richness) in the tunnel (*P* = .047 and .0007, respectively), but not in the open field (*P* = .72 and .72, respectively). Furthermore, in the tunnel, several pollinator-associated genera, such as *Hymenobacter* and *Massilla* were significantly more prevalent on unnetted flowers than on netted flowers. This was not the case in the open field (Fig. 3G and H).

## Discussion

In this study, we assessed the impact of agricultural intensification practices on the short-lived flower bacterial community. Among the factors characterizing cultivation intensification, the degree of sheltering, ranging from open-air to tunnel to greenhouse, showed the biggest influence on the flower microbiome, explaining 11% of community composition variability. We found that flower communities were less diverse in more controlled environments, while the variability between samples increased and prevalent, recurring core taxa were lost.

Consequently, despite greenhouse conditions being more constant and favorable for plant growth, the bacteria associated with the flowers on these plants did not form a steady, potentially beneficial community, and many open flower-associated taxa were lost in the more controlled environments. This indicates that flower-associated bacterial communities require a certain level of input from environmental bacteria, lacking in controlled environments, to reach a consistent composition.

Theoretically, these low-diversity communities are more prone to invasion [46], both beneficial and pathogenic. This was demonstrated in practice as we found that the beneficial bacterium *B. amyloliquefaciens* (the active ingredient of plant protection product Taegro®) had successfully colonized the majority of flowers when the bacteria were sprayed before flower opening of netted and unnetted flowers. This example demonstrates the potential of deliberately enriching microbiome-poor cultivation environments with key bacterial taxa to stabilize bacterial communities and improve plant health, especially by reintroducing taxa lost in controlled environments.

Among the taxa lost in more intensively managed systems are *Burkholderiaceae* 1, *Conyzicola* 1 and 2, *Microbacteriaceae* 1, *Micrococcaceae* 1, *Neorhizobium* 2, *Pseudomonas*_O 1, and *Sphingomonas* 1. Interestingly, all these were part of the core community of open-air cultivated strawberries but occurred significantly less in greenhouses and could no longer be identified as core taxa, showing that the naturally occurring functional community is heavily disturbed in sheltered strawberry flowers. Based on their prevalence and abundance, these taxa play an important role in the anthosphere community. Their disappearance in intensively managed systems suggests their use in restoration or biocontrol strategies. *Sphingomonas*, e.g. is a genus known to be a common member of the plant core microbiome and its benefits in plant growth promotion and pathogen suppression have been demonstrated previously [47–49]. Olimi et al. (2022) observed less *Sphingomonas* in diseased strawberries than in ripe and stored fruits and hypothesized this was linked to a disrupted state, potentially favoring opportunists or pathogenic taxa [47]. *Methylobacterium*, significantly more prevalent in open fields than tunnels, is not only a common phyllosphere member [47, 50, 51] but can also influence strawberry taste and odor by producing volatile organic compounds [52]. Also, Sangiorgio et al. (2021) linked management practices to raspberry fruit quality, possibly mediated by producing volatile organic compounds by, among other taxa, *Methylobacterium* and *Sphingomonas* [8]. Therefore, enriching cultivation environments with key taxa could affect fruit quality.

A second parameter that increases in more heavily managed systems is substrate-based cultivation. Our study demonstrates that independent of the effect of cultivation type, substrate-based cultivation reduced the bacterial inverse Simpson index in flowers compared to soil-based cultivation. Moreover, the R^2^-value of 0.04 computed via PERMANOVA analysis is an underestimation as substrate cultivation was positively associated with greenhouse cultivation. Similarly, applying plant protection products also positively correlated with sheltered cultivation and therefore its R^2^-value of 0.03 was underestimated. Moreover, it reduced the flower bacterial richness and inverse Simpson index in open fields and tunnels. These PPPs were mostly fungicides, based on Captan, Kresoxim-methyl, Fluopyram, Trifloxystrobin, Cyprodinil, and fludioxonil. Similarly, Wei et al. (2021) observed a decline in bacterial Shannon diversity on flowers in an open strawberry field after applying the fungicide azoxystrobin or bactericide oxytetracycline [53]. Such antimicrobial products can disrupt host-microbiome homeostasis, leaving the plant more susceptible to stressors [54]. Therefore, the use of chemical PPPs should be minimized and preventative biological augmentation should be considered, as prescribed by the integrated pest management directive, issued by the European Commission (https://food.ec.europa.eu/plants/pesticides/sustainable-use-pesticides/integrated-pest-management-ipm_en).

Moreover, field surface area significantly influenced flower diversity, albeit differently for sheltered and open cultivation types. The inverse Simpson index positively correlated with the strawberry patch surface area in open fields, especially organically maintained. Wei et al. (2021) observed a similar correlation between flower abundance and the floral microbiome in open-field strawberry patches, which they attributed to the larger source pool, the bigger resource availability to attract pollinators, and the intrinsic abundance of microbes that can colonize flowers through wind or rain events [53]. This positive richness-area relationship is a well-established concept in ecology [55]. However, in covered cultivation systems, the alpha diversity decreased with a larger surface area. This seemingly counterintuitive observation could be explained by the reduced dispersal of microbes through wind, rain, and pollinators in sheltered environments, limiting the source pool and levelling off species richness [55]. This corresponds with the generally poor and random flower microbiome we observed in greenhouses, where the likelihood of random flower inoculation decreases with an increasing number of flowers. This is further supported by the positive correlation between surface area and beta diversity in sheltered conditions, indicating that large flower populations are associated with a reduced presence of a recurring core community. Conversely, in open-field strawberry patches, increasing surface area was associated with greater similarity and the development of a core community. Additionally, a larger surface area in covered systems is often linked to more intensive agriculture and professionalization, reducing the diversity of cultivated crops and manual labor on the farm, while increasing hygiene regulation.

Flowers, especially those of strawberries, are short-lived structures [30]. Consequently, the bacterial communities inhabiting them do not result from long ecological succession processes as seen in the phyllosphere or rhizosphere [56]. Furthermore, nearly all strawberry flowers are visited by pollinators, which is integral to their reproductive process, and also introduces certain microbes [31]. In this study, several genera such as *Prevotella*, *Leuconostoc*, *Schwartzia*, *Staphylococcus*, and *Pseudomonas* were abundant on both flowers and foraging pollinators in the open field. At the same time, *Staphylococcus*, *Bacillus*, and *Pseudomonas* were abundant on both flowers and foraging pollinators in the greenhouse. This suggests that flowers may act as transmission hubs for certain bacteria [57]. Conversely, some taxa are specialized to pollinators such as *Gilliamella*, *Bombilactobacillus*, *Lactobacillus*, *Snodgrassella*, and *Frischella*. These were significantly more present on foraging bees than on flowers in the open field, indicating bacterial transfer from forager to flower without subsequent growth or establishment on the flowers. Similarly, Legein et al. (2022) found *Gilliamella* and *Snodgrassella* as core microbiome members of commercially reared bumblebees foraging in the greenhouse, but these bacteria were far less abundant on strawberry leaves in the same greenhouse [20].

Excluding large pollinators from flowers did not significantly change the flower microbiome composition, suggesting that large pollinator visits did not directly contribute much to the presence of anthosphere bacteria under the tested circumstances. However, pollinator foraging did increase bacterial alpha diversity on flowers in tunnels, but not in the open field. This could be explained by open-field flowers accessing flowers through other mechanisms such as wind or rain. Wei et al. (2021) observed a positive correlation between bee visitation and bacterial alpha diversity in open-field strawberry flowers [53]. Netting the flowers in our experiment possibly did not prevent pollinator-associated taxa from entering through other means, such as wind or smaller vectors, indicating that large pollinators contribute to bacterial dispersal onto flowers in addition to different mechanisms.

The proportion of unique ASVs shared between flowers and pollinators was significantly higher in the tunnel than in the open field, aligning with our finding that anthosphere microbial communities are less stable in more intensively managed systems, and thus more susceptible to introduced microbes. This highlights the potential benefits of introducing microbes in intensively managed systems, possibly via pollinators. While microbial dispersal through pollinators, or entomovectoring, has been done previously with a positive effect on pathogen susceptibility [58–60], it has yet to be explored with microbes selected for their role in stabilizing the anthosphere microbiome. The potential of rewilding strategies, including bee vectoring with microbes such as *Sphingomonas*, *Pseudomonas*, and *Methylobacterium*, to influence disease resistance and fruit quality requires further investigation to provide growers with accurate guidance. When determining optimal cultivation practices, it is essential to balance trade-offs among yield, year-round production, PPP usage and regulations, and fruit quality.

## Conclusion

This study highlights the impact of agricultural practices on the diversity and stability of the microbial communities of short-lived flowers. In intensively managed systems like greenhouses and tunnels, the bacterial diversity on the flowers significantly decreased, core taxa were lost, and the variability among flowers increased, compared to less intensively managed systems like open fields and wildflowers. Moreover, the microbial diversity lost in greenhouses overlapped with members of the core community found in open-field flowers, making these taxa promising candidates for enriching microbe-poor cultivation systems. In addition to these effects of sheltering, we found that using plant protection products, substrate-based cultivation, and the size of the cultivation area further reduced the bacterial richness, resulting in an altered flower community composition. Furthermore, using pollinator-exclusion experiments, we revealed that foraging pollinators in sheltered environments significantly aid in shaping the flower microbiome, but much less so in open fields. This underscores that pollinators could be used as vectors to introduce microbes in intensively managed agricultural systems to enhance plant health and fruit quality. Overall, our study underscores the importance of understanding the interactions between agricultural practices and the flower microbiome. These insights are essential for developing targeted restoration strategies that support plant health and optimize fruit quality.

## Supplementary Material

Supplementary_figure_1_ycaf026

Supplementary_figure_2_ycaf026

Supplementary_figure_3_revised_ycaf026

Supplementary_figure_4_revised_ycaf026

Supplementary_figure_5_22_05_2025

Supplementary_figure_6_revised_ycaf026

Supplementary_figure_7_revised_ycaf026

Supplementary_figure_8_revised_ycaf026

Supplementary_figure_9_ycaf026

Supplementary_figure_10_ycaf026

Supplementary_information_21_03_Update_ycaf026

## Data Availability

This study’s sequencing data and metadata were made available under study accession number PRJEB79350 in the European Nucleotide Archive. Sample information can be retrieved via this link: https://www.ncbi.nlm.nih.gov/biosample?linkname=bioproject_biosample&from_uid=1179818 or with the NCBI E-utilities (https://www.ncbi.nlm.nih.gov/books/NBK25500/) using the following command: esearch -db bioproject -query “PRJEB79350” | elink -target biosample | efetch -format docsum | xtract -pattern DocumentSummary -block Attribute -element Attribute > Sabofleur_metadata.tsv.
