# Supplementary material for: Agricultural practices and pollinators modulate the anthosphere microbiome
Source: ISME Commun. 2025 Feb 12;5(1):ycaf026. doi: 10.1093/ismeco/ycaf026 (PMC12118460; doi:10.1093/ismeco/ycaf026)
Supplement: Supplementary_figure_1_ycaf026 [file supplementary_figure_1_ycaf026.pdf]

Universiteit Antwerpen

EMÜ

SABOFLEUR

Lab of applied Microbiology and Biotechnology, UAntwerp, Belgium
Chair of plant health, Estonian University of life sciences, Estonia

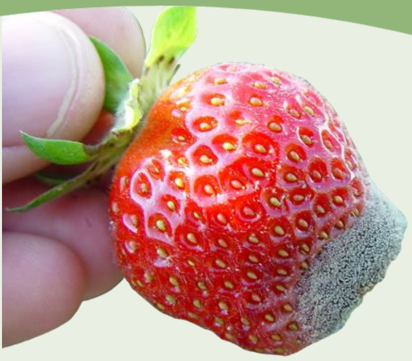

What can be found in the box?

- Gloves for sterile work
- Ethanol wipes to clean your scissors
- 6 tubes with and without washing buffer (see markings on tubes)
- Retour ticket + cover for free return shipping

How do I start?

1. Put on the **gloves** and clean them with the ethanol wipes. Avoid contact with the flowers at every step.
2. Take a pair of scissors and clean them with the ethanol wipes.
3. Choose **three strawberry plants** with at least **four flowers each** to donate.
4. Using the clean scissors, cut of **three flowers** at the stem and transfer them to **one of the provided tubes with washing buffer**. Avoid touching the flowers, or having them fall on the floor. Do this by holding the tube just below the flower when cutting.
5. Following the same directions, cut **one more flower** from the same plant and transfer it to **one of the provided tubes without washing buffer**.
6. Repeat for the other 2 plants to obtain a total of six filled tubes: three tubes with washing buffer and three flowers each + three tubes without buffer containing one flower each (see figure).
7. **Seal the tubes tightly** and place them in the cover for shipping.
8. After collection you can fill out the **form** on the overleaf. Place this fill-in sheet in the cover for shipping.
9. Close the box and apply the return ticket and send by mail. We have prepaid the postal fees for you, so no need to pay.

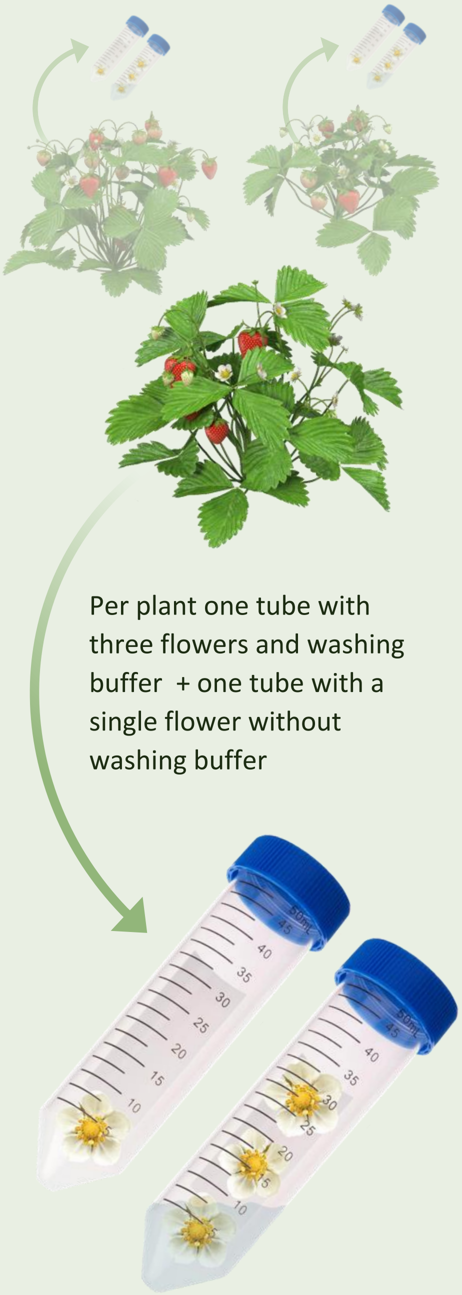

Universiteit Antwerpen

EMU

Fill-in form

Name person or company: ....

Address: ....

Indicate: ○ Hobby grower

○ Professional grower

Indicate: ○ Organic cultivation

○ Non-organic cultivation

Date of sample collection: ..../........../2023

Indicate: ○ Greenhouse

○ Open field

○ Tunnels

○ Other:

Indicate: ○ Soil

○ Substrate

○ Heated

○ Unheated

Surface area of the plot: ....

Cultivar: ....

Indicate: ○ Natural pollination

○ Mechanical pollination

○ Pollinating insects, namely: .... (species)

Recently treated? ○ No

○ Yes, on: ..../........../2023

Treated with: ....

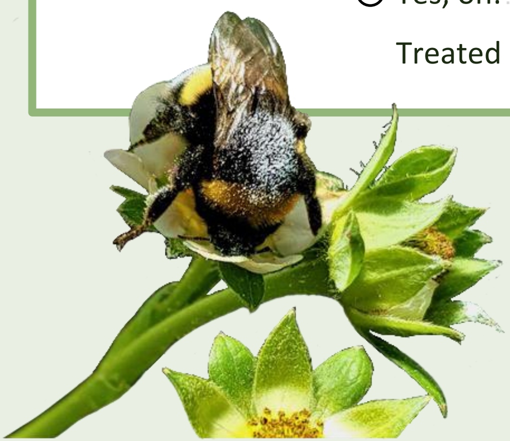

Dear strawberry lovers and growers,

If you're reading thbis letter, it means you have agreed to join the fight against grey mould! Amazing! Thanks to your strawberry flowers, scientists at the University of Antwerp can look for good bacteria that sabotage this harmful fungus on the flower (hence **Sabofleur**)!

What happens to my donated flowers?

Once the flowers reach the lab, they are washed to rinse off all bacteria. Subsequently, all bacteria on these flowers are examined. The good bacteria are picked out and we try to find out whether they can help prevent the growth grey mould.

Why am I being asked to work through the manual?

- In the lab, we will be looking for good bacteria on the flowers, because of this, contact with the flowers must be avoided as much as possible. Therefore, you are asked to work with gloves so that your own bacteria are not accidentally transferred.
- The flowers are cut off and placed in a washing solution. This solution serves to wash the bacteria off the flower.
- The fill-in sheet gives the scientists an idea about the differences between the types of cultivation and practices.

Can I find out more about the results?

In addition to supporting this scientific project, you will receive a personal results report telling you which bacteria are present on your own strawberry flowers. This way you can actively see what you have contributed to!

Thanks to you we can fight against grey mould!

Thank you very much for your commitment!

Kind regards,
The lab team of the University of Antwerp and
the Estonian University of Life Sciences

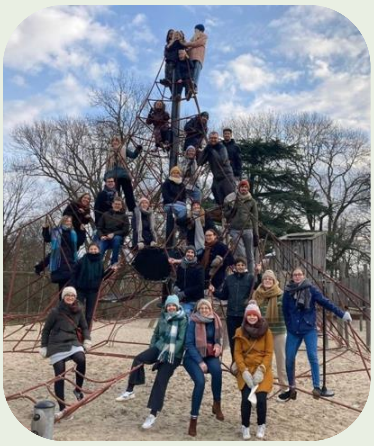

If you should have any questions, do not hesitate to send to
jari.temmermans@uantwerpen.be
