# Supplementary figures and images for: Agricultural practices and pollinators modulate the anthosphere microbiome
Source: ISME Commun. 2025 Feb 12;5(1):ycaf026. doi: 10.1093/ismeco/ycaf026 (PMC12118460; doi:10.1093/ismeco/ycaf026)

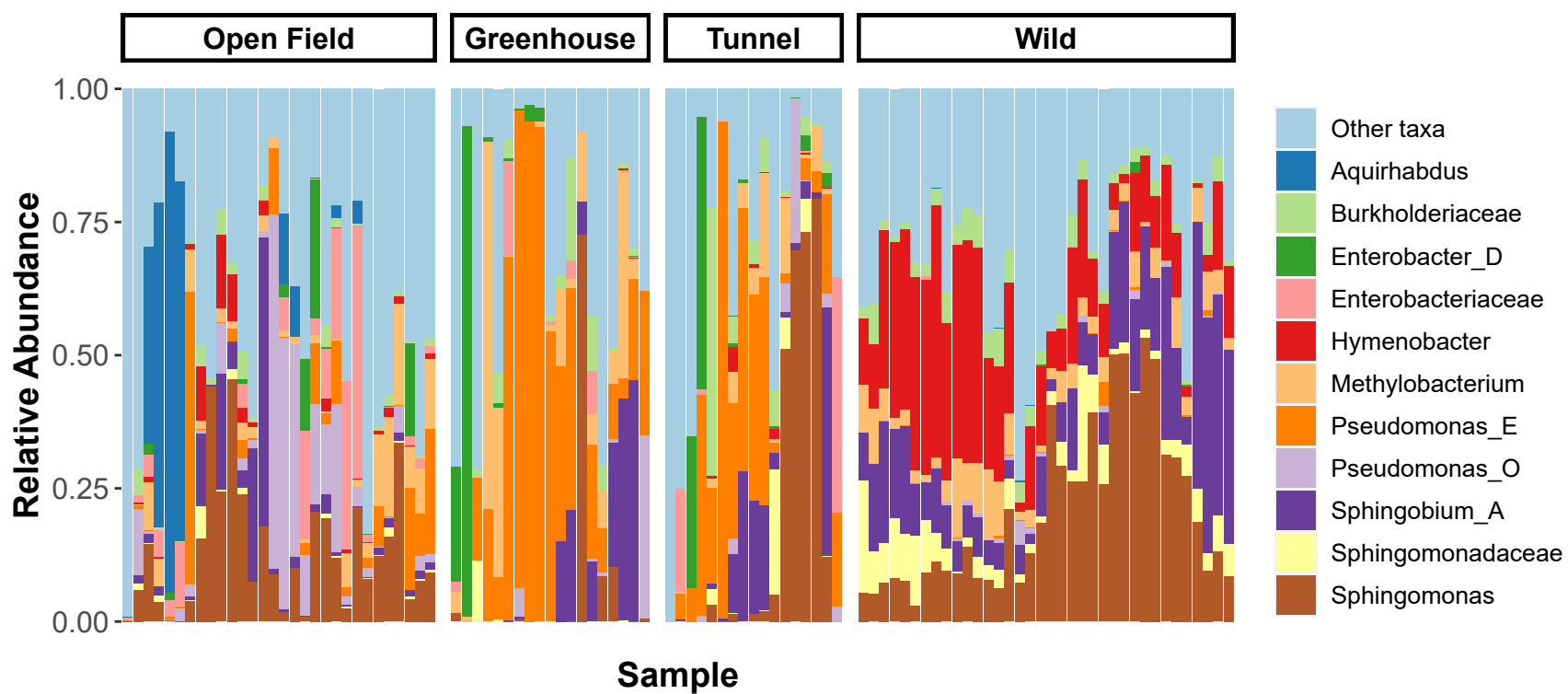

Supplement: Supplementary_figure_2_ycaf026 [file supplementary_figure_2_ycaf026.pdf]

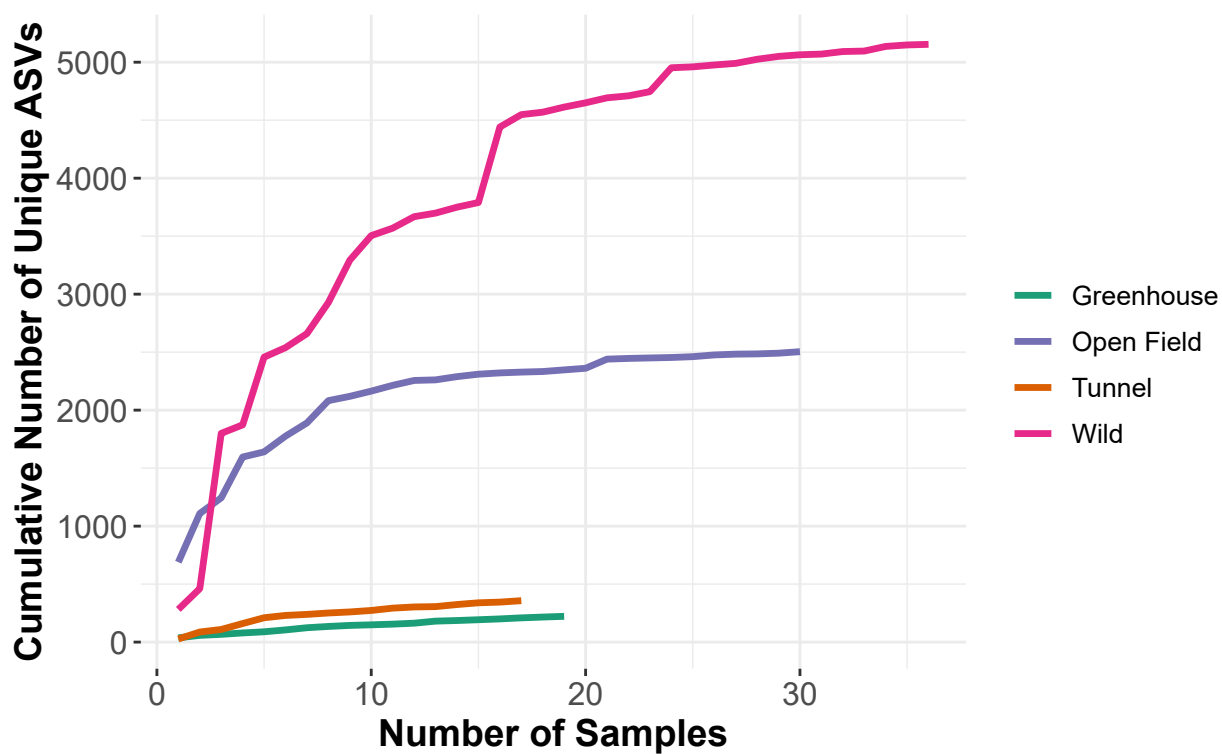

Supplement: Supplementary_figure_3_revised_ycaf026 [file supplementary_figure_3_revised_ycaf026.pdf]

# Beta Diversity

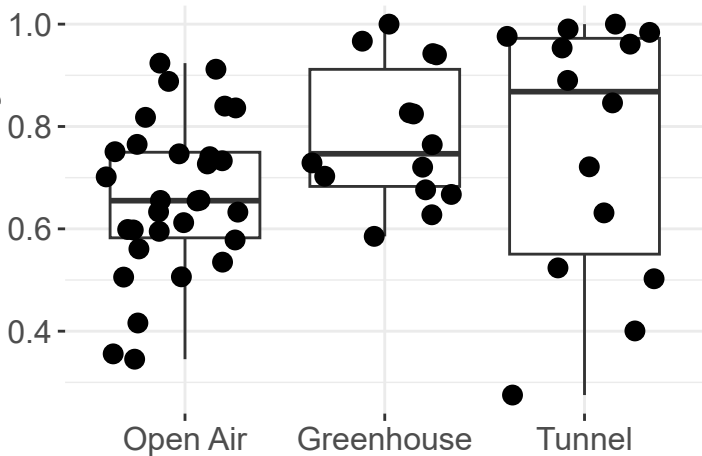

Supplement: Supplementary_figure_4_revised_ycaf026 [file supplementary_figure_4_revised_ycaf026.pdf]

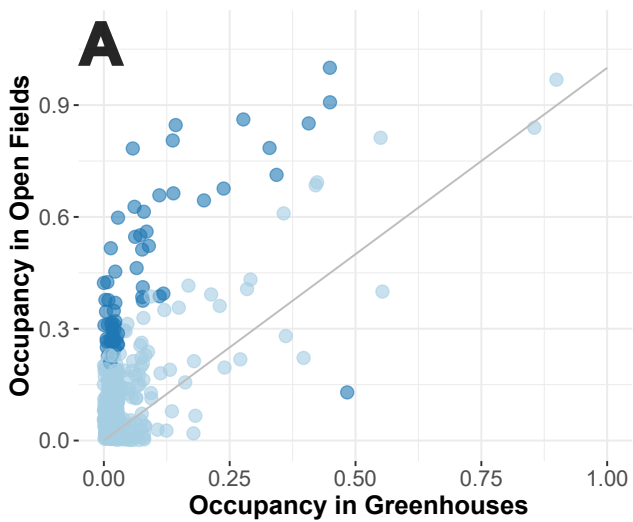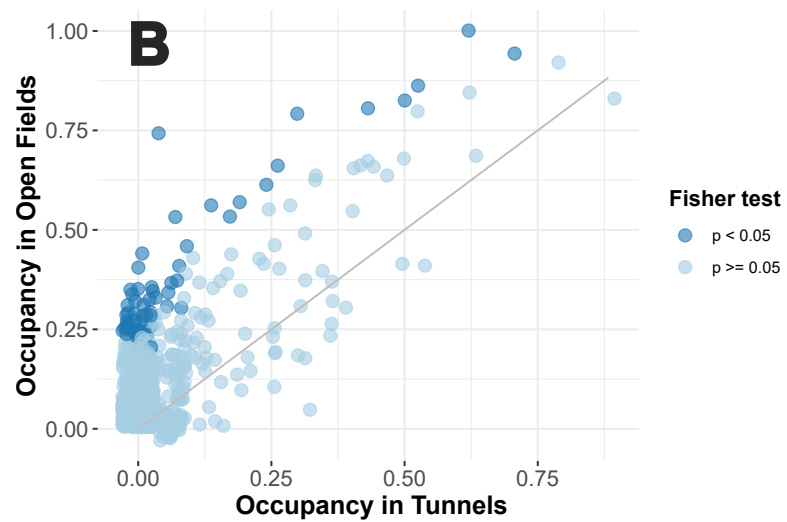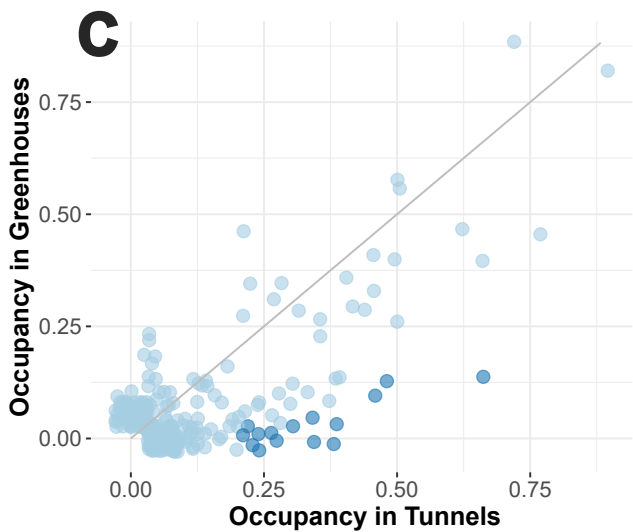

Supplement: Supplementary_figure_5_22_05_2025 [file supplementary_figure_5_22_05_2025.pdf]

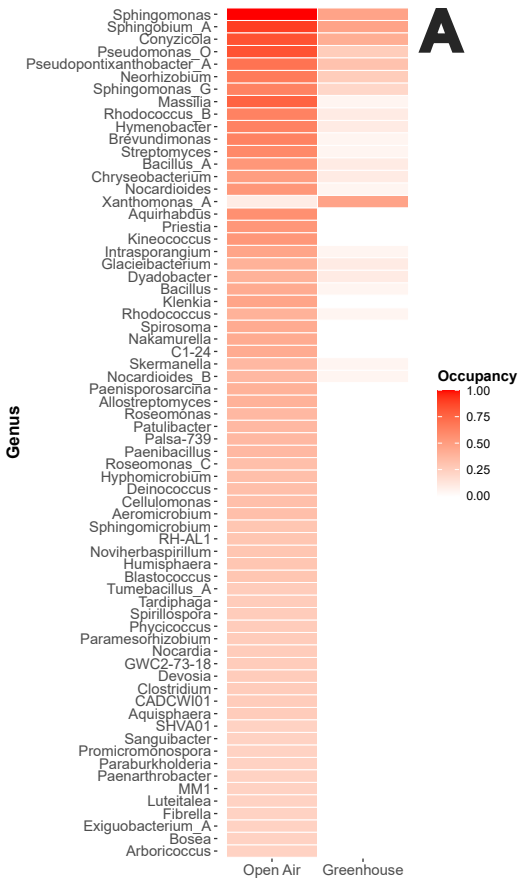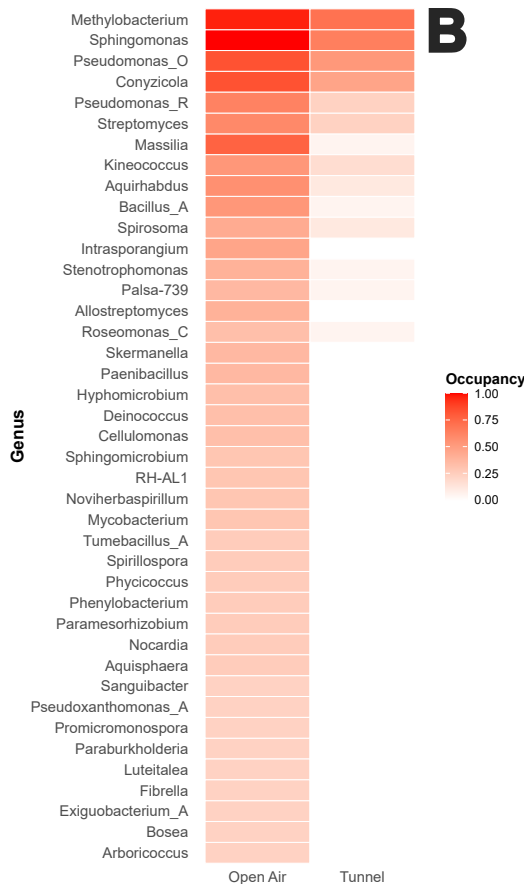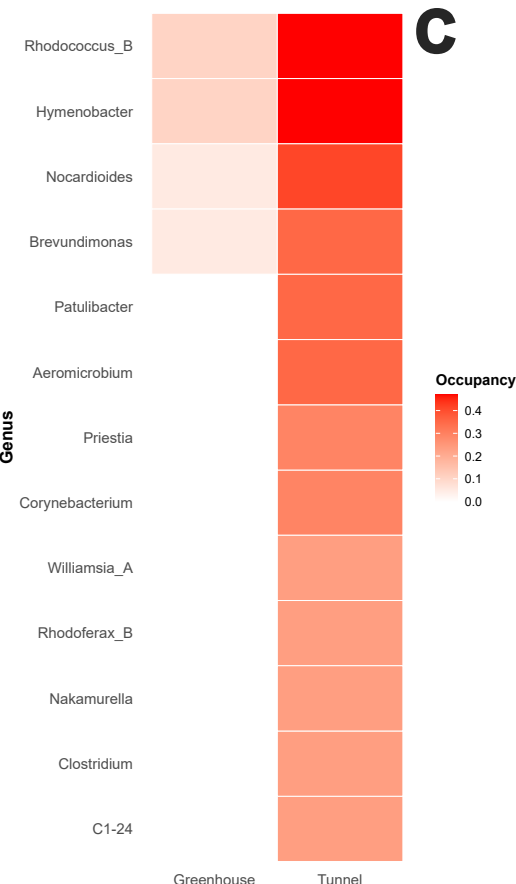

Supplement: Supplementary_figure_6_revised_ycaf026 [file supplementary_figure_6_revised_ycaf026.pdf]

**A****log(Inverse Simpson)**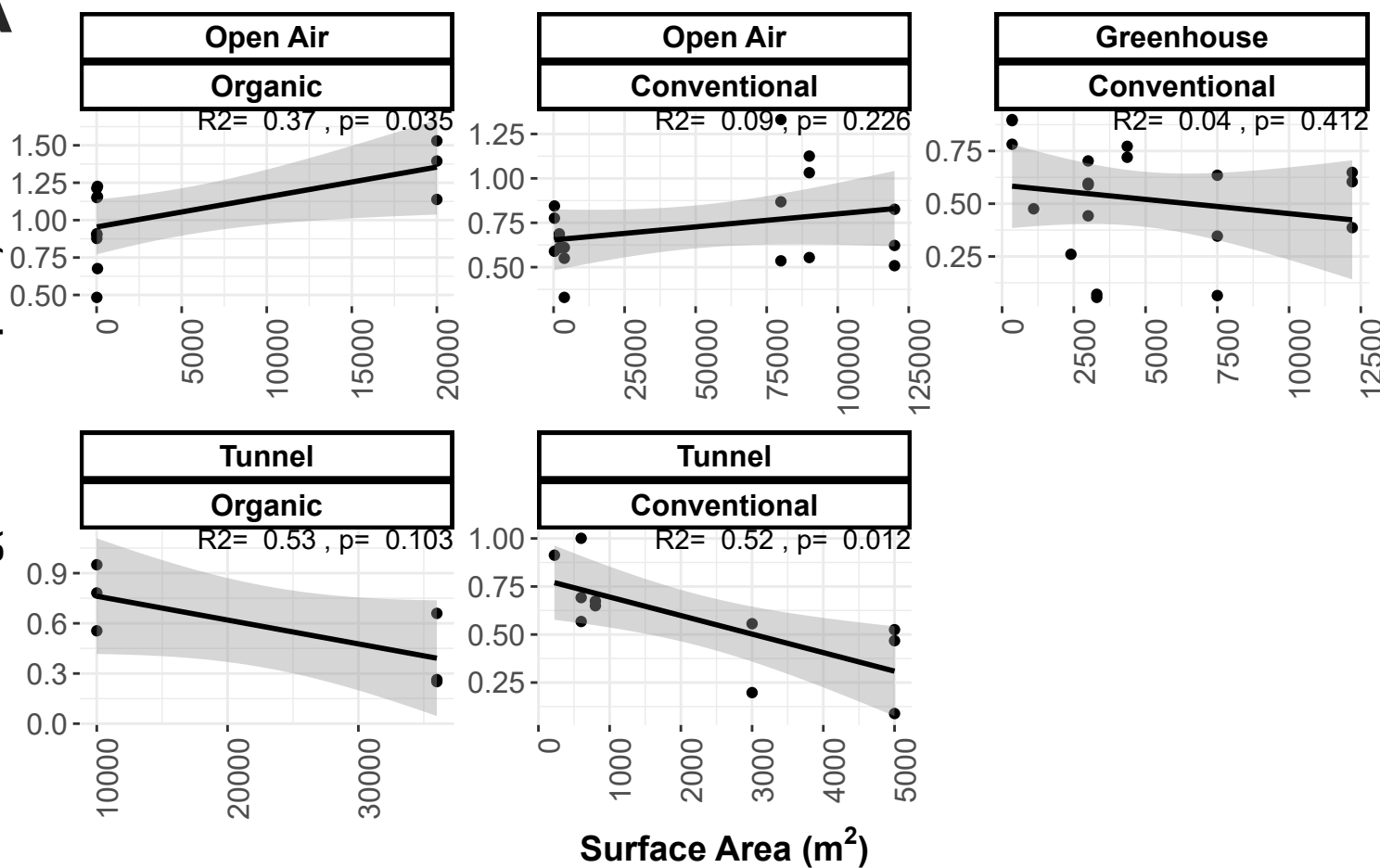**B****Beta Diversity**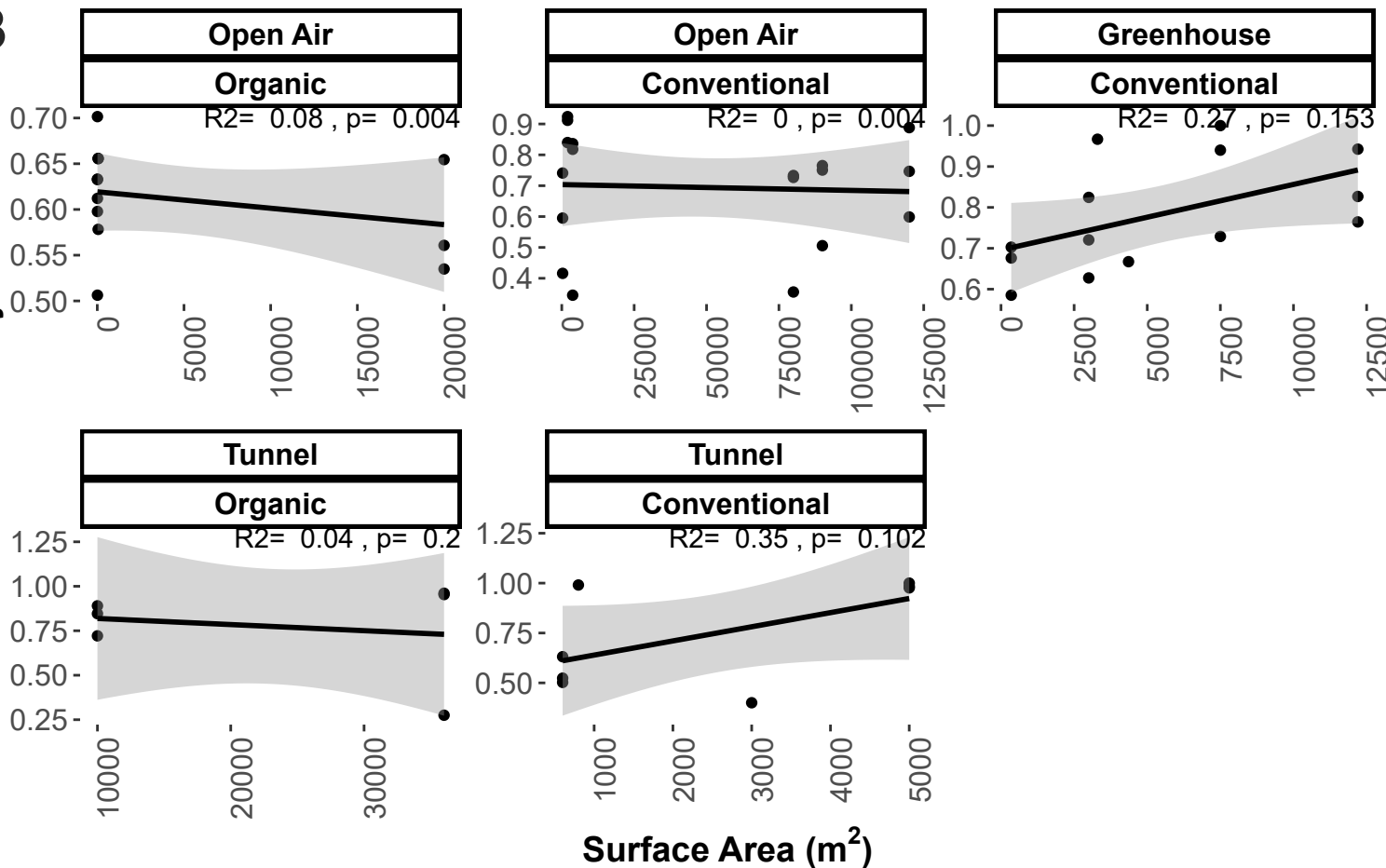

Supplement: Supplementary_figure_7_revised_ycaf026 [file supplementary_figure_7_revised_ycaf026.pdf]

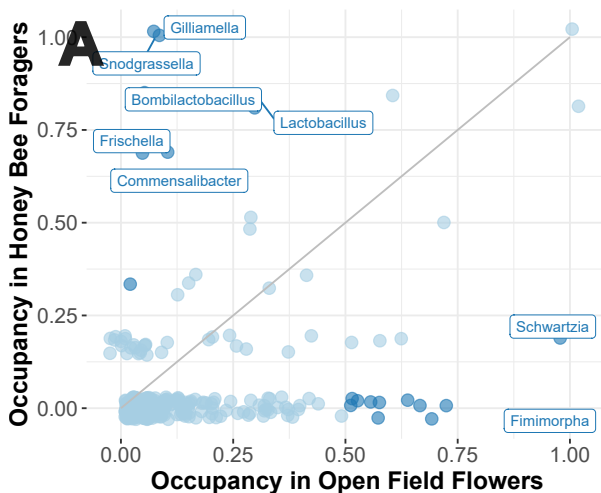

Fisher test

- a  $p < 0.05$
- a  $p \geq 0.05$

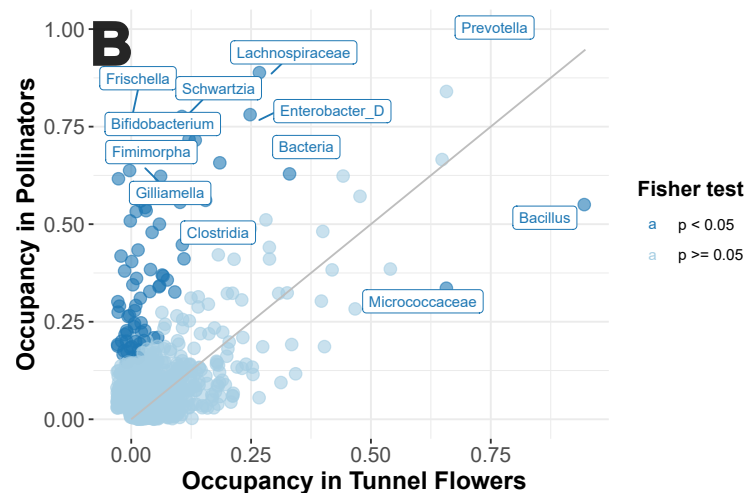

Fisher test

- a  $p < 0.05$
- a  $p \geq 0.05$

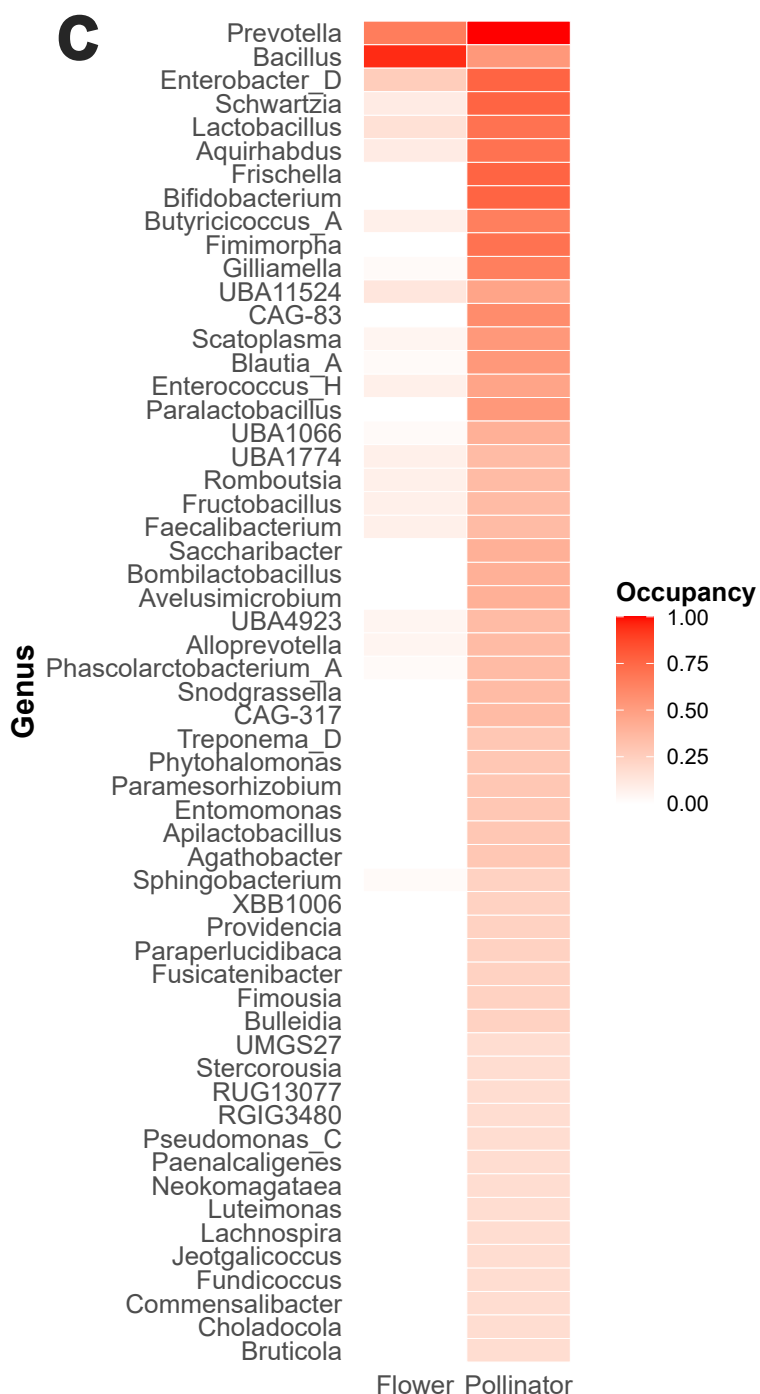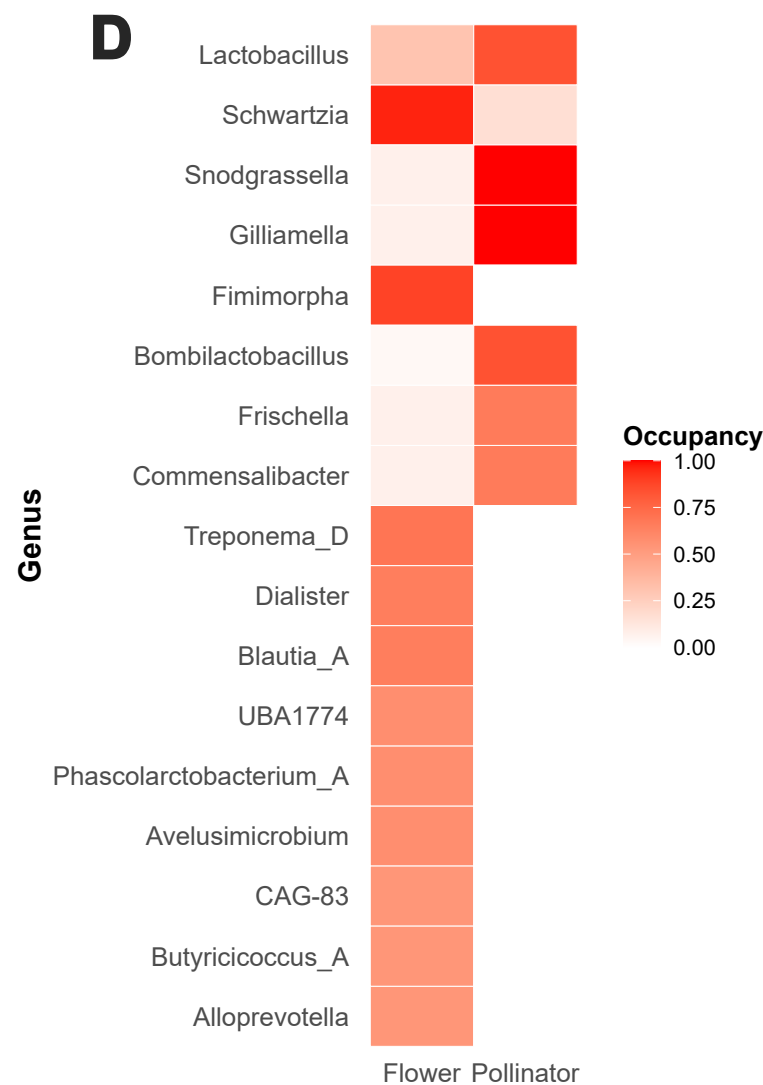

Supplement: Supplementary_figure_8_revised_ycaf026 [file supplementary_figure_8_revised_ycaf026.pdf]

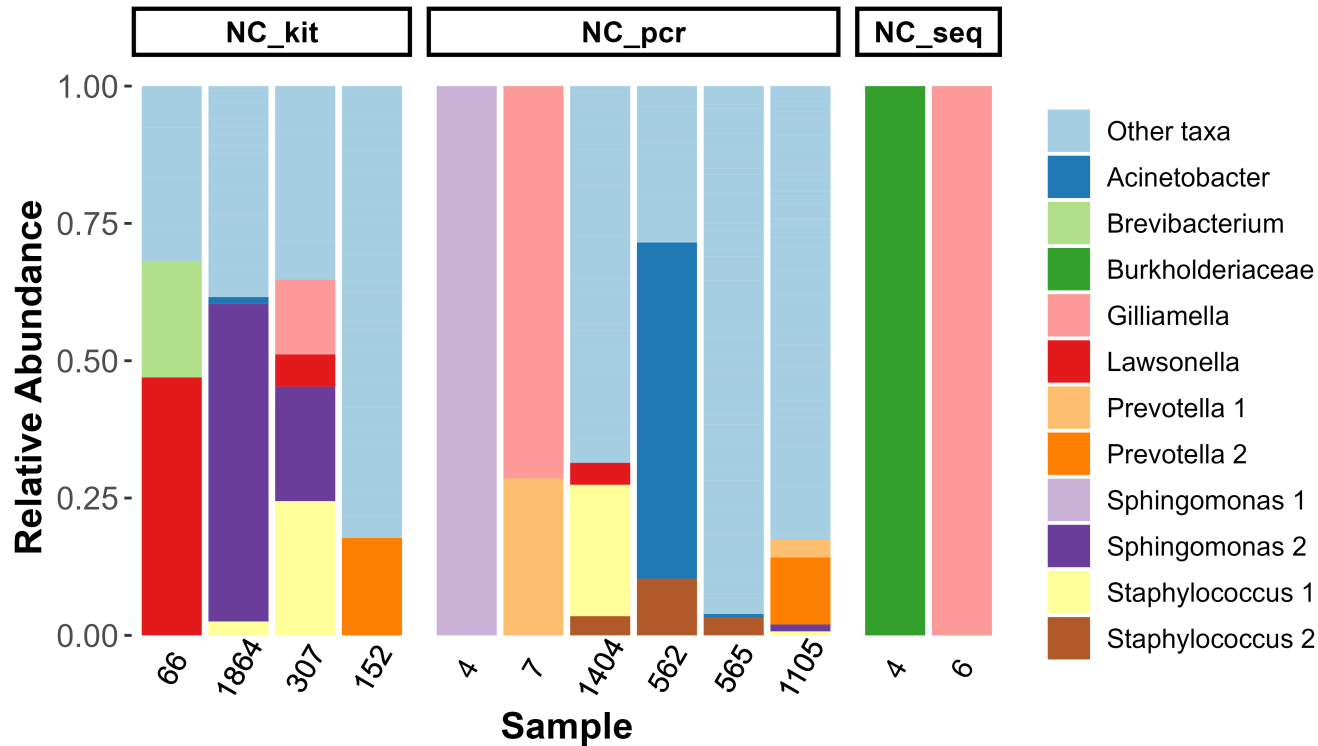

Supplement: Supplementary_figure_9_ycaf026 [file supplementary_figure_9_ycaf026.pdf]

**A**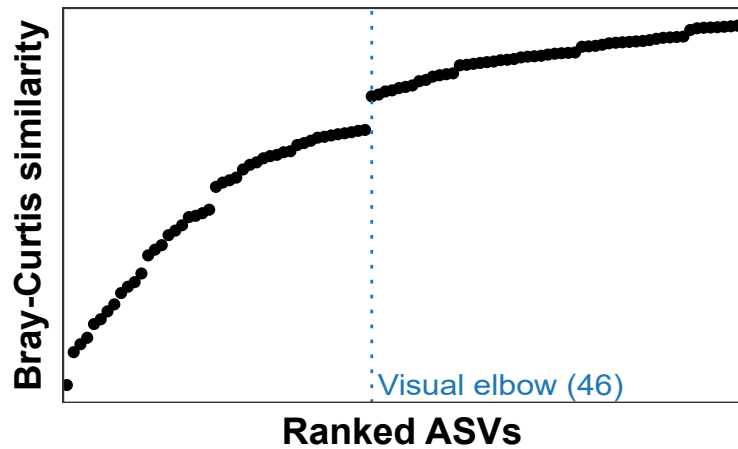**B**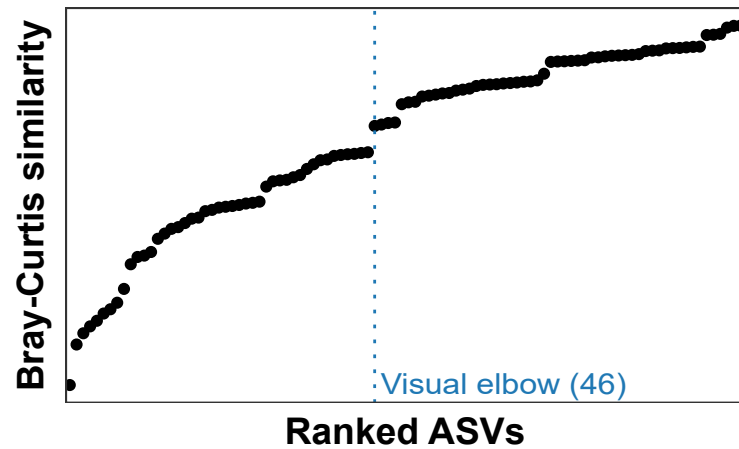**C**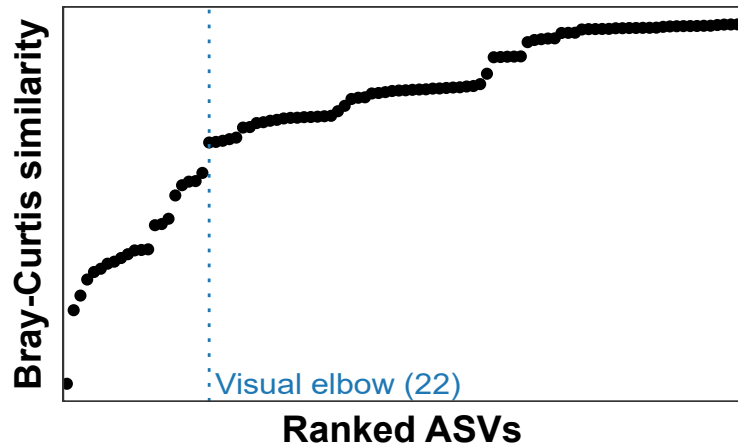**D**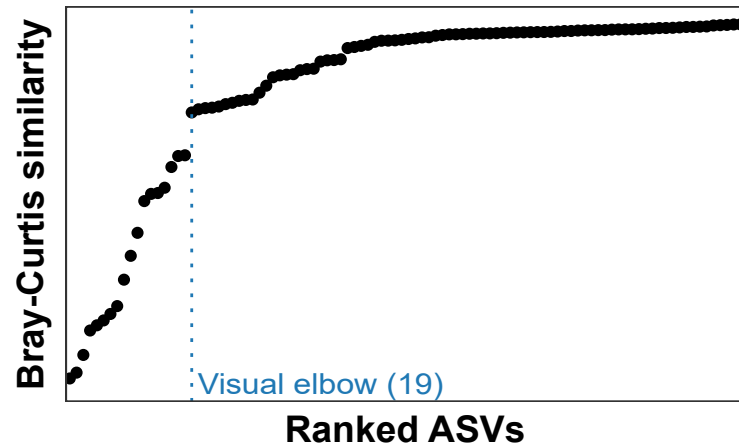

Supplement: Supplementary_figure_10_ycaf026 [file supplementary_figure_10_ycaf026.pdf]
