# Supplementary material for: Agricultural practices and pollinators modulate the anthosphere microbiome
Source: ISME Commun. 2025 Feb 12;5(1):ycaf026. doi: 10.1093/ismeco/ycaf026 (PMC12118460; doi:10.1093/ismeco/ycaf026)
Supplement: Supplementary_information_21_03_Update_ycaf026 [file supplementary_information_21_03_update_ycaf026.docx]

# Supplementary information


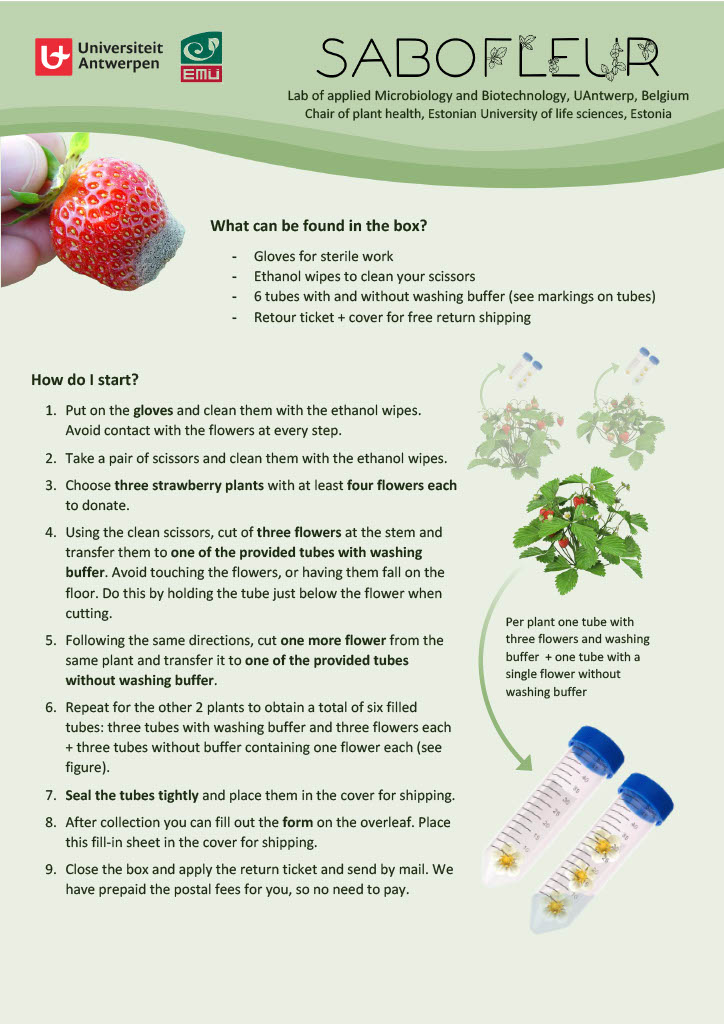


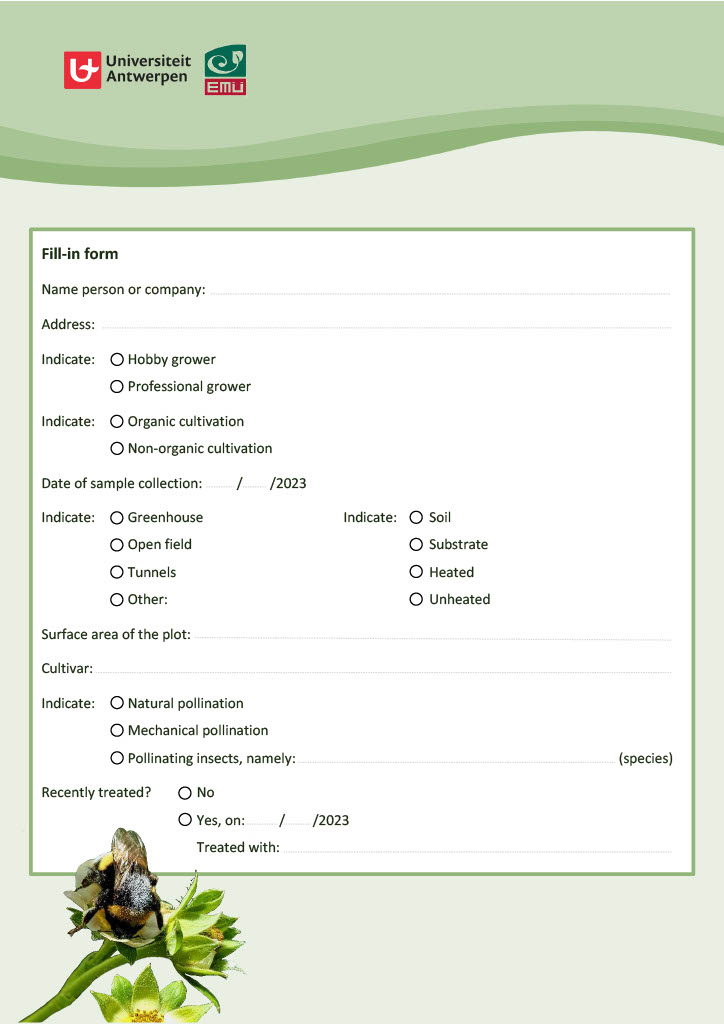


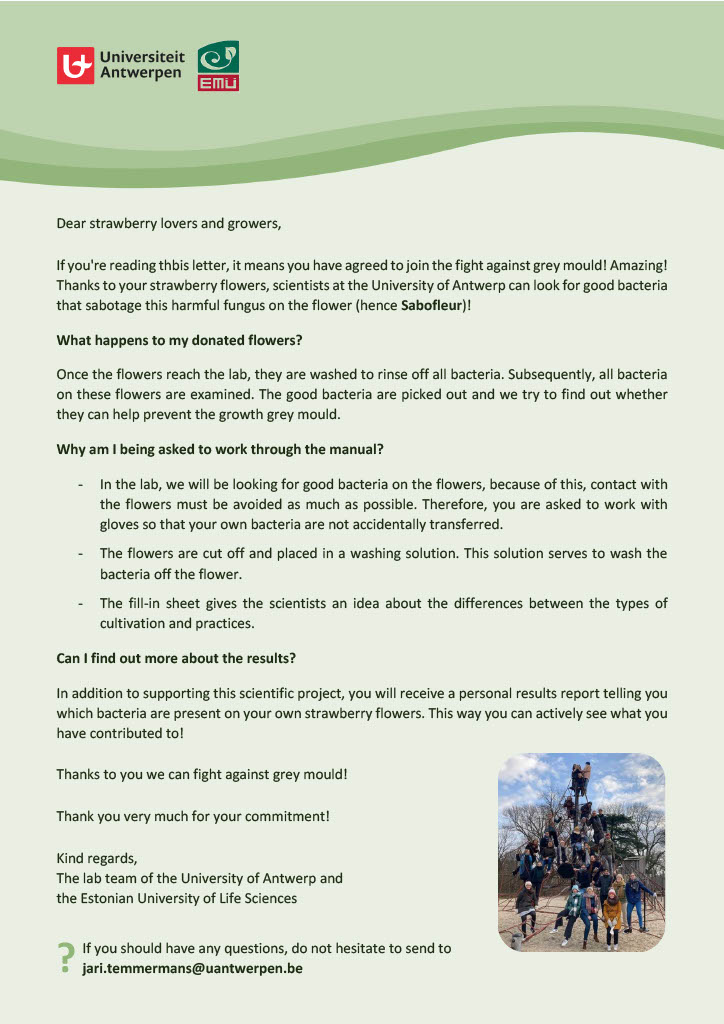


Supplementary Figure 1: Instruction manual for participating growers. Translated from Dutch.


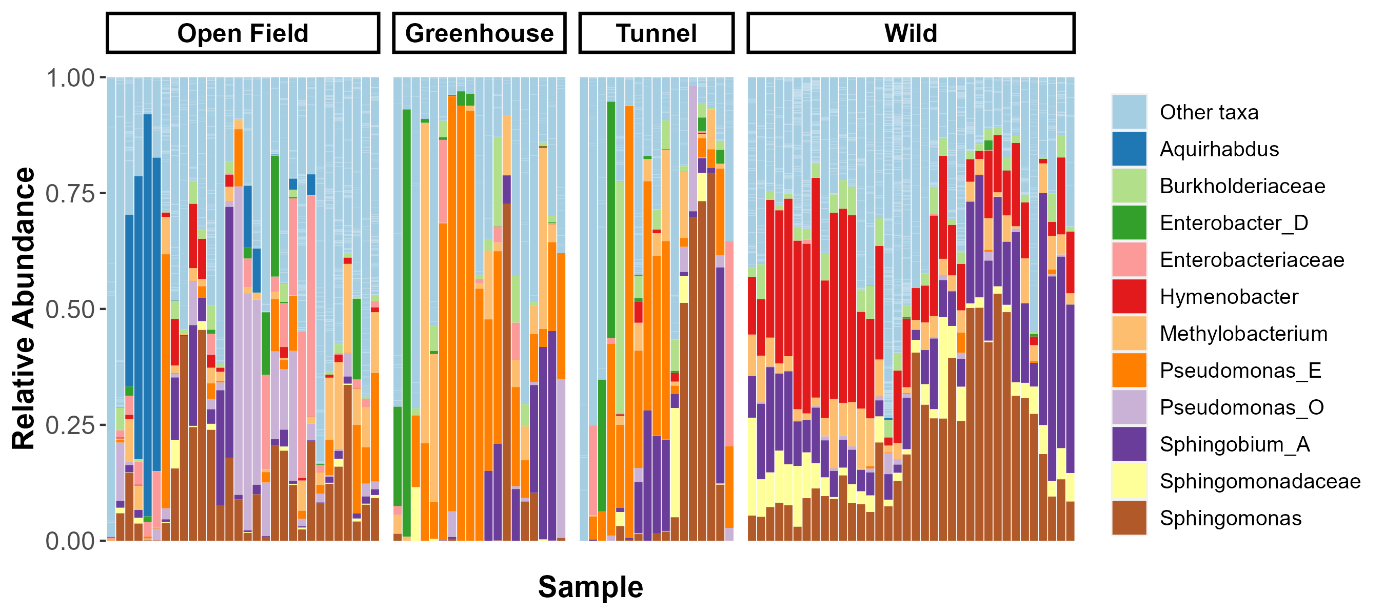


Supplementary Figure 2: Relative abundance of the 10 most abundant taxa in the strawberry flowers sampled during the Sabofleur project, based on a genus level. ‘Other taxa’ refers to the number of samples with a different genus.


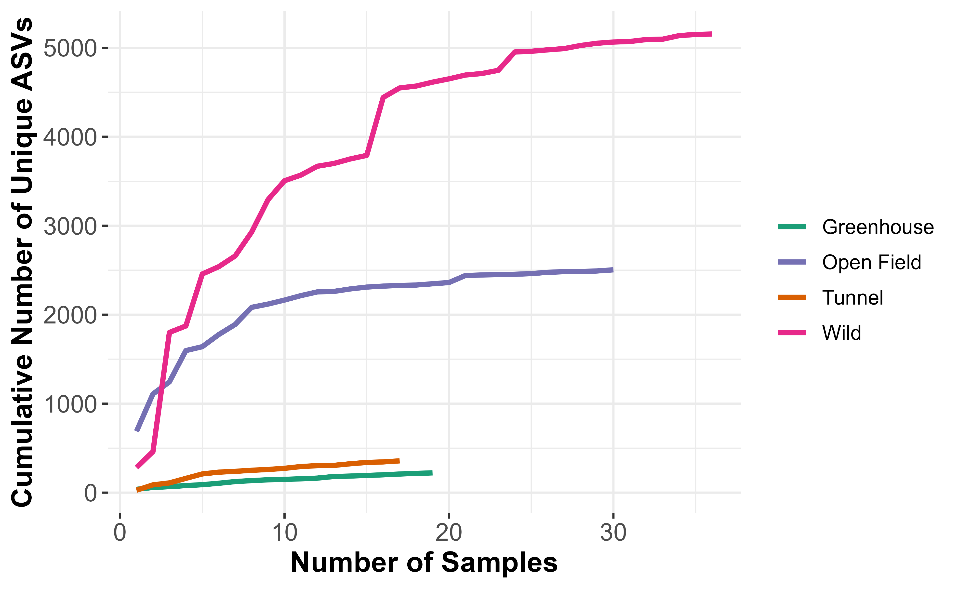


Supplementary Figure 3: Cumulative number of unique ASVs as a function of the number of strawberry flower samples obtained from four different cultivation types after ordering the samples from high to low abundances in every cultivation category.


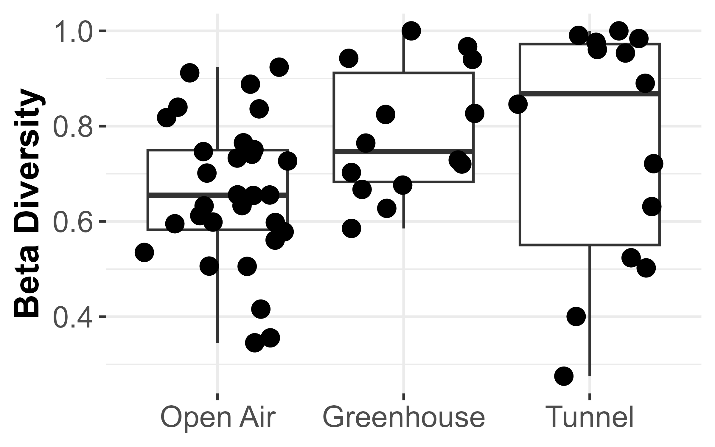


Supplementary Figure 4: Intra-farmer beta diversity. Similarly to between the cultivation types: variability is higher in covered cultivation systems.

Supplementary Table 1: Core ASVs across all cultivation types, determined using the occupancy-abundance elbow approach developed by Shade et al. (2019) (39). If the ASV is a core ASV in the respective cultivation type, it is depicted with “✔”.

| Taxon Name | Greenhouse | Tunnel | Open Air | Wild |
| --- | --- | --- | --- | --- |
| *Pseudomonas*_E 1 | ✔ | ✔ | ✔ |  |
| *Pseudomonas*_E 2 | ✔ | ✔ | ✔ |  |
| *Sphingobium*_A 1 | ✔ | ✔ | ✔ | ✔ |
| *Rhizobiaceae* 1 | ✔ | ✔ | ✔ | ✔ |
| *Enterobacter*_D 2 | ✔ |  |  |  |
| *Pseudomonas*_E 3 | ✔ | ✔ |  |  |
| *Sphingomonas* 1 | ✔ | ✔ | ✔ | ✔ |
| *Methylobacterium* 1 | ✔ | ✔ | ✔ | ✔ |
| *Methylobacterium* 3 | ✔ | ✔ | ✔ |  |
| *Pseudomonas*_O 1 | ✔ | ✔ | ✔ | ✔ |
| *Duganella* | ✔ |  |  |  |
| *Acinetobacter* 1 | ✔ |  |  |  |
| *Ensifer*_A | ✔ | ✔ |  |  |
| *Xanthomonas*_A | ✔ |  |  |  |
| *Methylobacterium* 2 | ✔ | ✔ | ✔ | ✔ |
| *Burkholderiaceae* 3 | ✔ | ✔ | ✔ | ✔ |
| *Enterobacter*_D 1 | ✔ | ✔ | ✔ |  |
| *Methylobacterium* 5 | ✔ |  | ✔ | ✔ |
| *Sphingomonas*_G 1 | ✔ | ✔ | ✔ | ✔ |
| *Sphingobium*_A 2 |  | ✔ | ✔ | ✔ |
| *Bacillus* 1 |  | ✔ |  |  |
| *Neorhizobium* 1 |  | ✔ |  |  |
| *Rhodococcus*_B 1 |  | ✔ | ✔ |  |
| *Methylobacterium* 4 |  | ✔ | ✔ | ✔ |
| *Burkholderiaceae* 7 |  | ✔ |  | ✔ |
| *Glacieibacterium* 1 |  | ✔ |  | ✔ |
| *Burkholderiaceae* 1 |  | ✔ | ✔ | ✔ |
| *Pedobacter* 2 |  | ✔ |  |  |
| *Priestia* |  | ✔ | ✔ |  |
| *Staphylococcus* 1 |  | ✔ |  |  |
| *Microbacteriaceae* 1 |  | ✔ | ✔ |  |
| *Patulibacter* 1 |  | ✔ |  |  |
| *Aquirhabdus* 1 |  |  | ✔ |  |
| *Enterobacteriaceae* 1 |  |  | ✔ |  |
| *Micrococcaceae* 1 |  |  | ✔ |  |
| *Conyzicola* 1 |  |  | ✔ | ✔ |
| *Streptomyces* 1 |  |  | ✔ |  |
| *Conyzicola* 2 |  |  | ✔ |  |
| *Luteibacter* 1 |  |  | ✔ |  |
| *Enterobacteriaceae* 2 |  |  | ✔ |  |
| *Pseudomonas*_O 3 |  |  | ✔ |  |
| *Massilia* 3 |  |  | ✔ |  |
| *Bacillus*_A 1 |  |  | ✔ |  |
| *Rhizobiaceae* 2 |  |  | ✔ | ✔ |
| Pseudomonas_O 2 |  |  | ✔ |  |
| C1-24 1 |  |  | ✔ |  |
| *Massilia* 4 |  |  | ✔ |  |
| *Massilia* 2 |  |  | ✔ | ✔ |
| *Methylobacterium* 10 |  |  | ✔ |  |
| *Rhizobiaceae* 3 |  |  | ✔ | ✔ |
| *Rhodococcus* 1 |  |  | ✔ |  |
| *Sphingomonadaceae* 1 |  |  | ✔ | ✔ |
| *Sphingobium*_A 6 |  |  | ✔ | ✔ |
| *Pseudomonas*_R 2 |  |  | ✔ |  |
| *Pseudopontixanthobacter*_A 1 |  |  | ✔ | ✔ |
| *Neorhizobium* 2 |  |  | ✔ |  |
| *Streptomycetaceae* 1 |  |  | ✔ |  |
| *Methylobacterium* 7 |  |  | ✔ | ✔ |
| *Streptomyces* 5 |  |  | ✔ |  |
| *Sphingomonas* 2 |  |  |  | ✔ |
| *Sphingobium*_A 3 |  |  |  | ✔ |
| *Hymenobacter* 1 |  |  |  | ✔ |
| *Hymenobacter* 2 |  |  |  | ✔ |
| *Hymenobacter* 3 |  |  |  | ✔ |
| *Hymenobacter* 4 |  |  |  | ✔ |
| *Hymenobacter* 5 |  |  |  | ✔ |
| *Hymenobacter* 6 |  |  |  | ✔ |
| *Aurantimonas* 1 |  |  |  | ✔ |
| *Sphingomonas*_M 1 |  |  |  | ✔ |
| *Hymenobacter* 11 |  |  |  | ✔ |
| *Sphingomonadaceae* 6 |  |  |  | ✔ |
| *Hymenobacter* 10 |  |  |  | ✔ |
| *Pedobacter* 3 |  |  |  | ✔ |
| *Sphingomonadaceae* 7 |  |  |  | ✔ |
| *Comamonas*_F 1 |  |  |  | ✔ |
| *Massilia* 1 |  |  |  | ✔ |
| *Hymenobacter* 12 |  |  |  | ✔ |
| *Sphingobium*_A 7 |  |  |  | ✔ |
| *Sphingobium*_A 5 |  |  |  | ✔ |
| *Brevundimonas* 1 |  |  |  | ✔ |
| *Sphingomonadaceae* 9 |  |  |  | ✔ |
| *Dyadobacter* 1 |  |  |  | ✔ |
| *Methylobacterium* 11 |  |  |  | ✔ |
| *Hymenobacter* 17 |  |  |  | ✔ |
| *Sphingobium*_A 8 |  |  |  | ✔ |
|  |  |  |  |  |


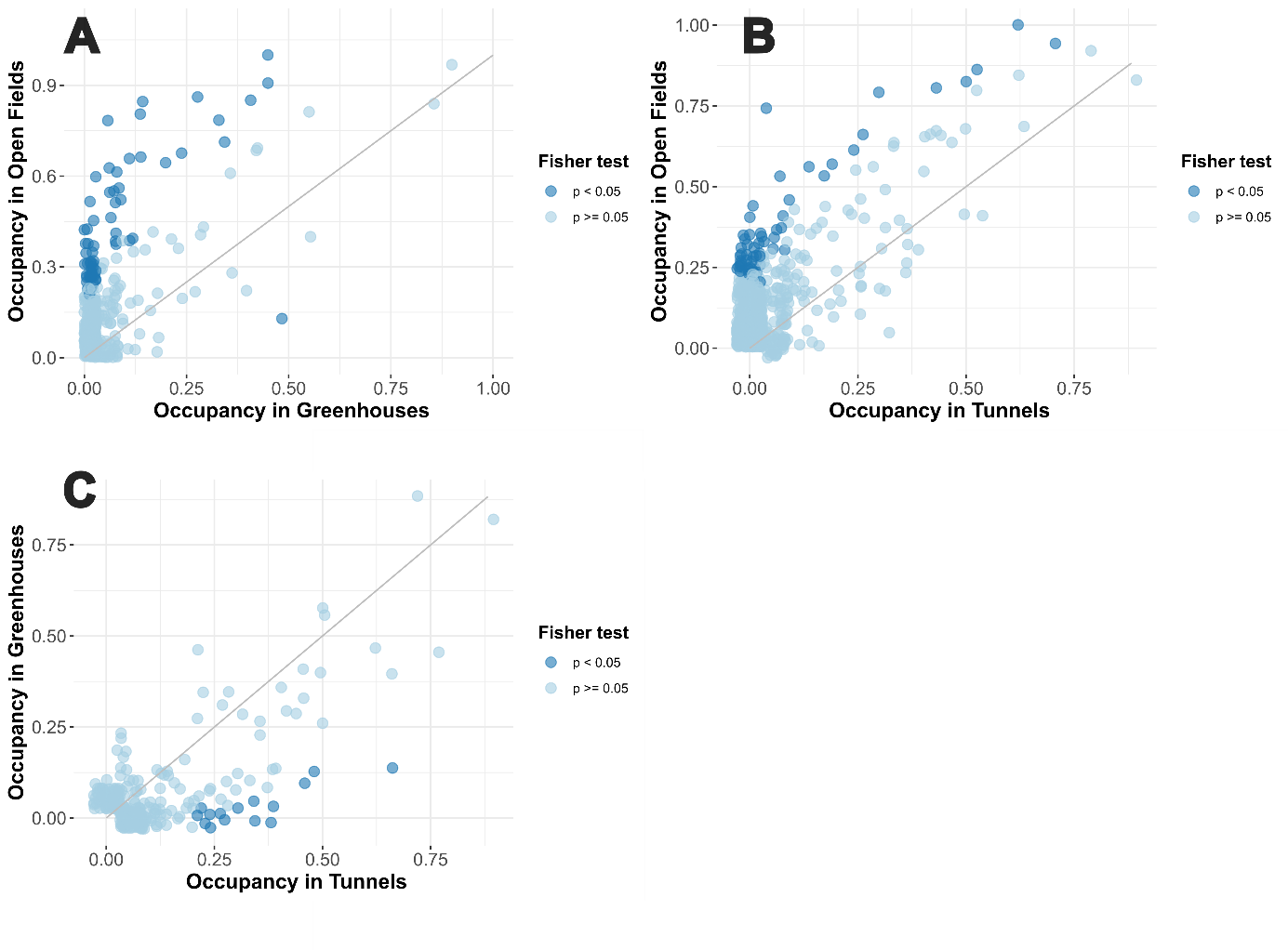


Supplementary Figure 5: Occupancy-occupancy plots for the different commercial cultivation types. A: Open fields vs greenhouses.B: Open fields vs Tunnels. C: Greenhouses vs Tunnels. Taxa occurring significantly more in one cultivation type based on Fisher’s test are depicted in dark blue.


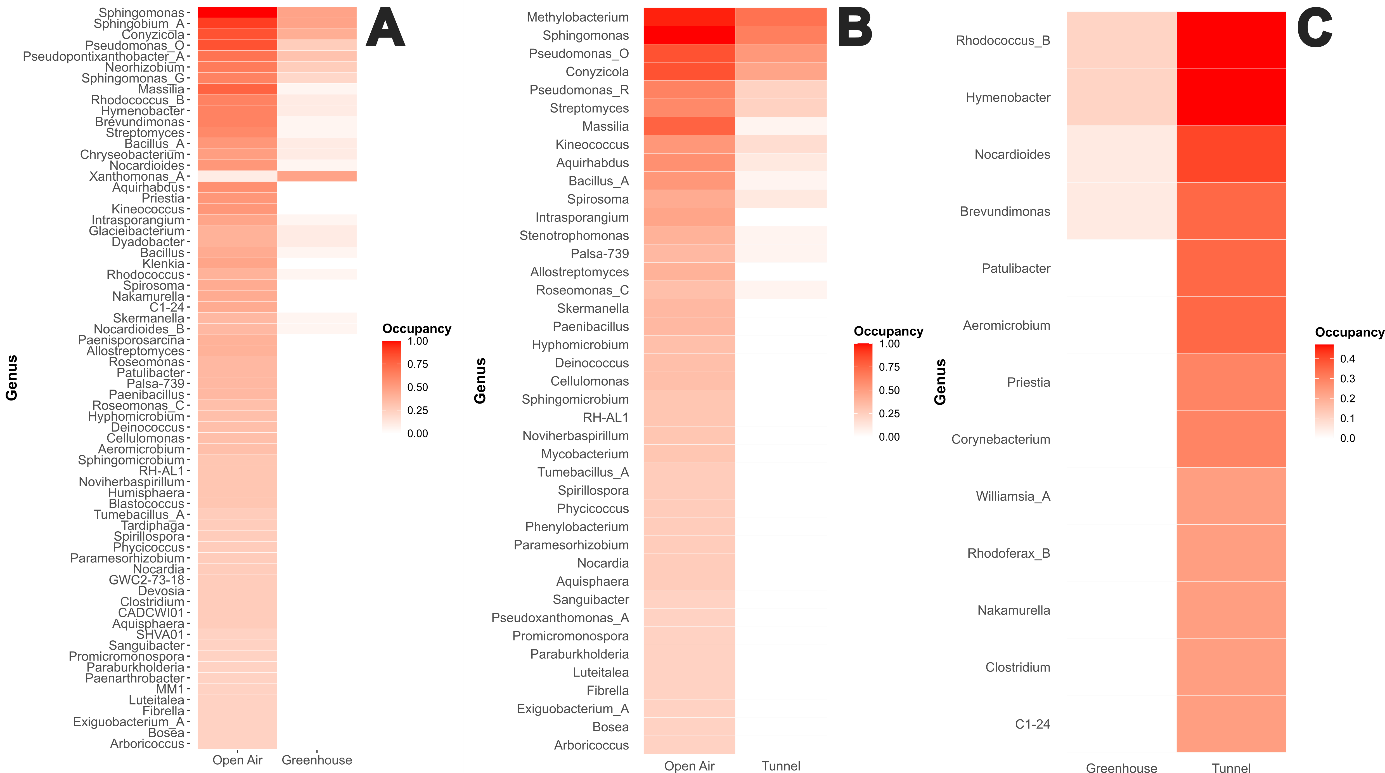


Supplementary Figure 6: Differential abundance for all taxa, significantly more prevalent in one cultivation type over another according to a pairwise Fischer test.


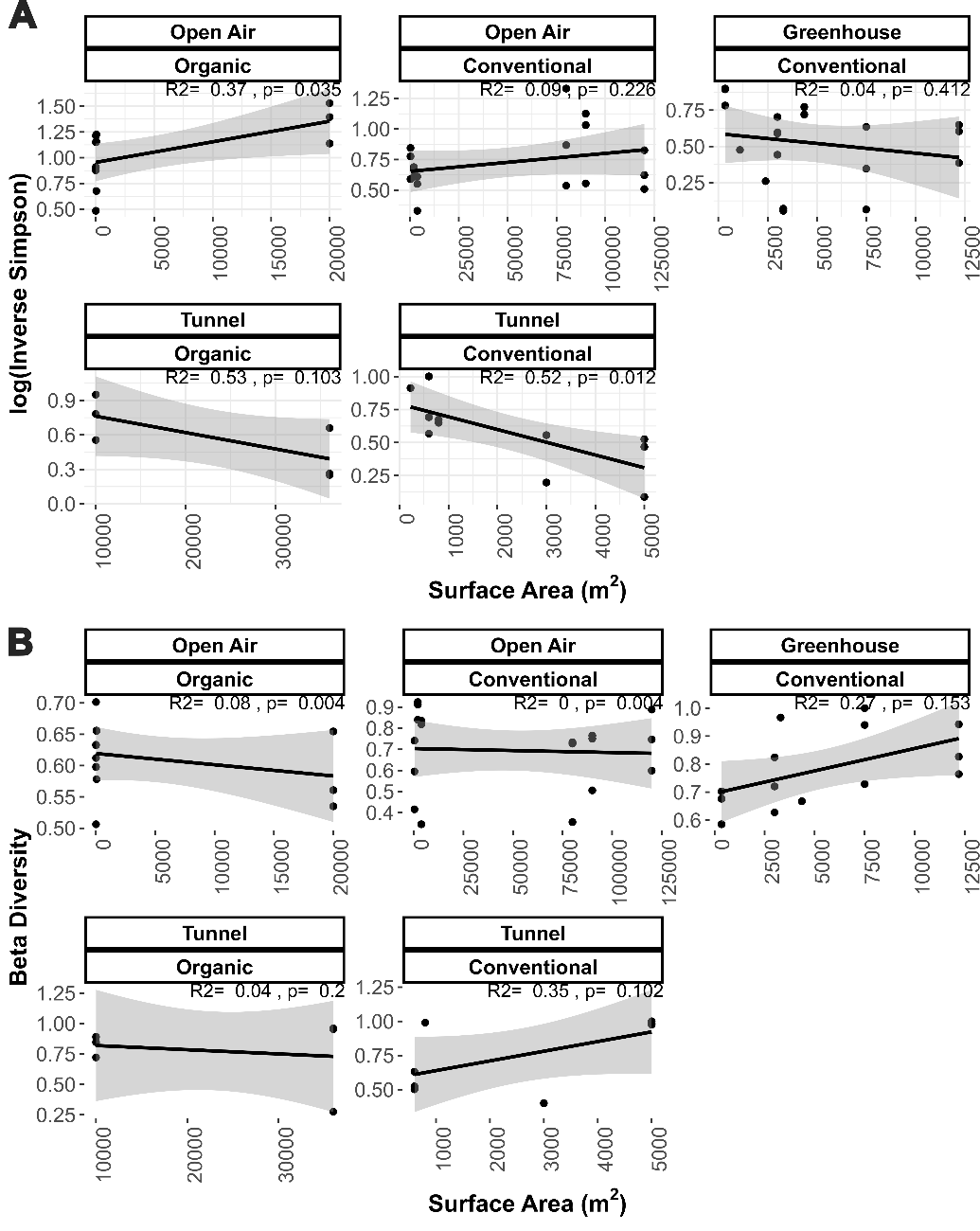


Supplementary Figure 7: A: Log(Inverse Simpson Index) in function of surface area. The p-values are calculated using a glm-model after subsetting for cultivation type and organic vs conventional cultivation. B: Within farm beta diversity in function of the surface area of the strawberry field. The p-values were calculated using constrained analysis of principal coordinates (CAP), followed by ANOVA after subsetting for cultivation type and organic vs conventional cultivation. Linear trendlines and 95% confidence interval were added.


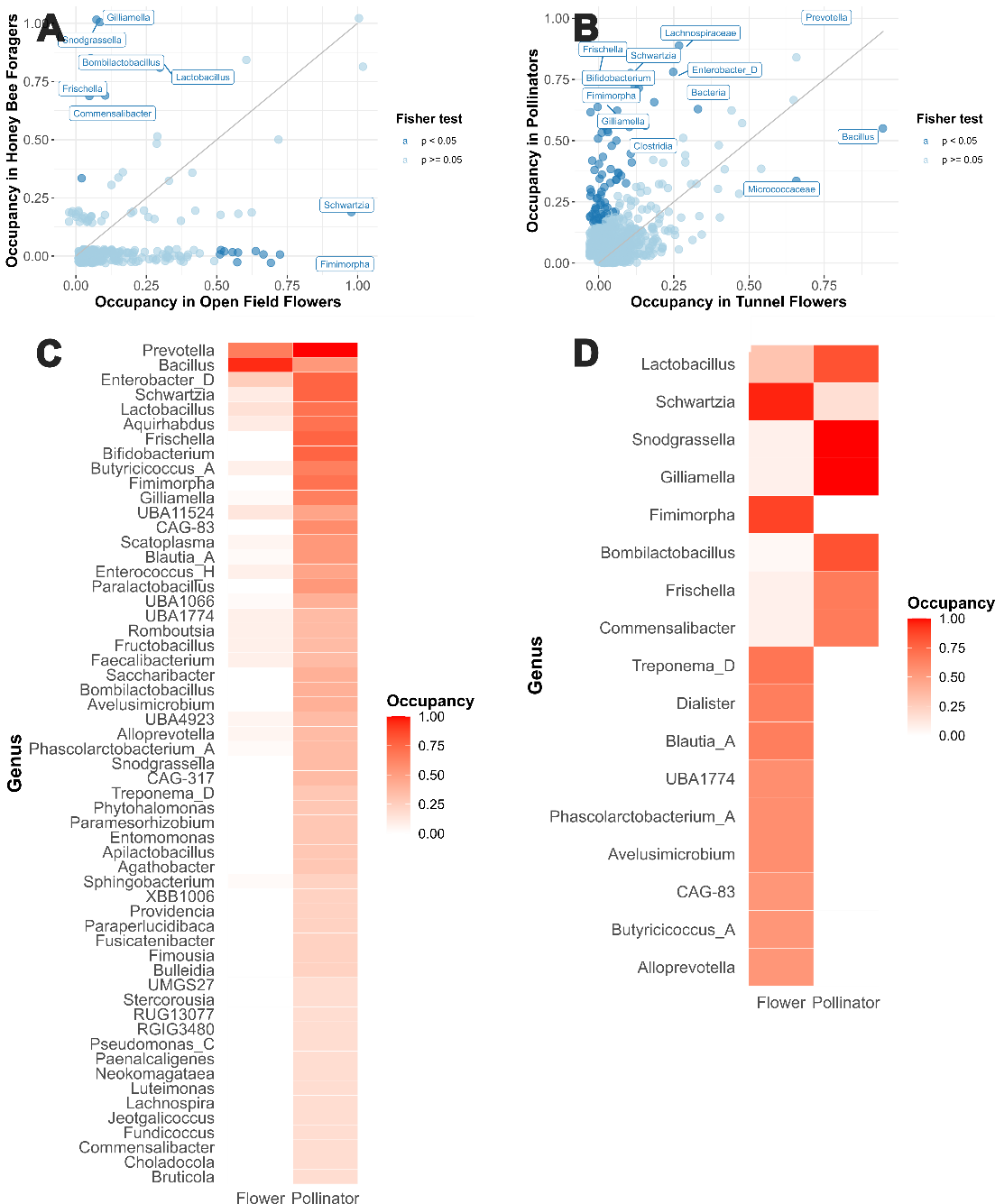


Supplementary Figure 8: A) + B) Occupancy-occupancy plots for both pollinator exclusion experiments. Open field flowers vs honeybee foragers (A) and tunnel flowers vs pollinators (B). C) + D) Differential abundance for all taxa, significantly more prevalent in flowers or pollinators in the open field (C) or the tunnel (D), according to a pairwise Fischer test.


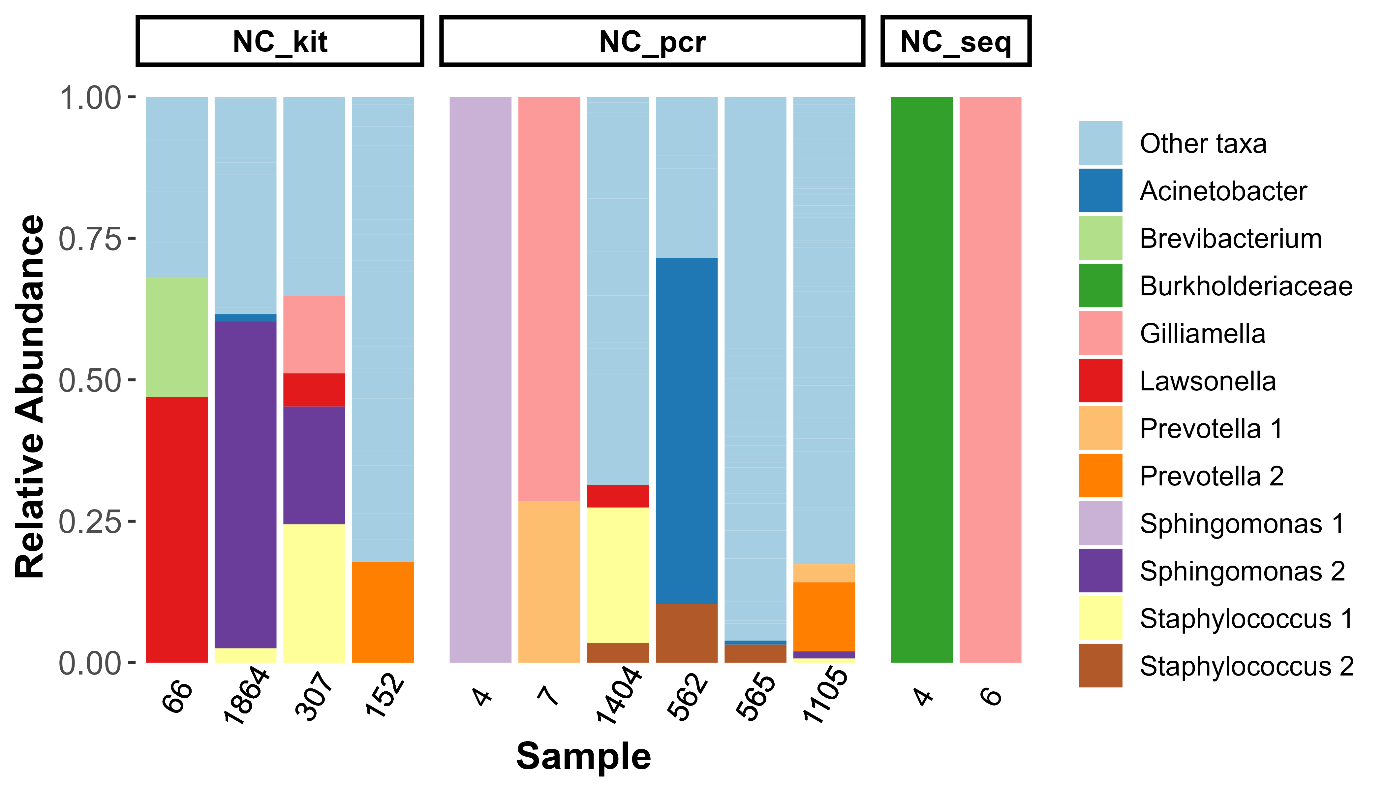


Supplementary Figure 9: Relative abundance of Extraction kit blanks (NC_kit), PCR blanks (NC_pcr), and sequencing blanks (NC_seq). Below each bar, the total number of read counts for that respective blank sample is depicted. The other blanks were empty.


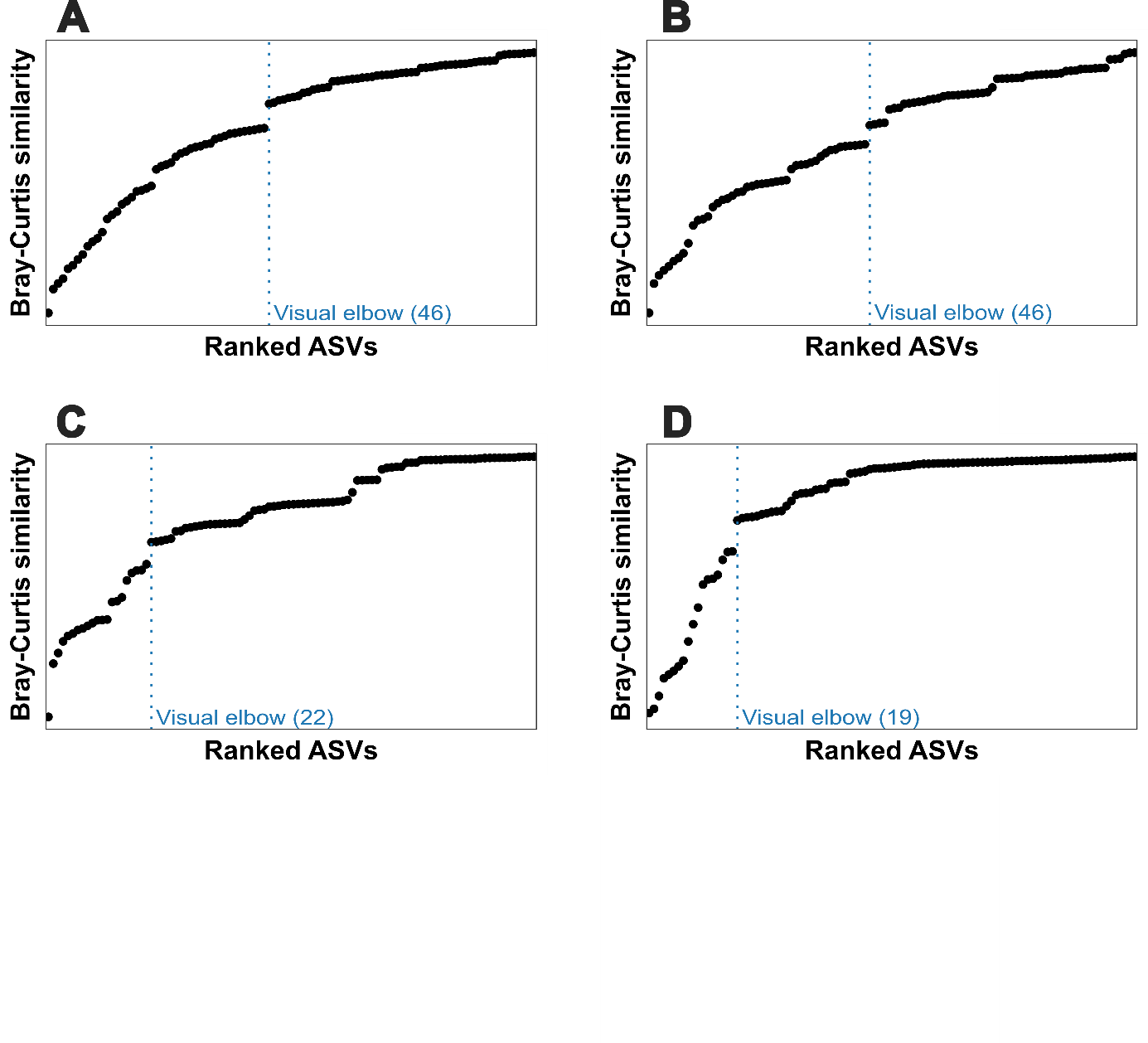


Supplementary Figure 10: Elbow method from Shade et al (2019) (39), employed to determine the core taxa per cultivation type. Elbows were determined based on Bray-Curtis similarity on a visual basis for Wild (A), Open Air (B), Tunnel (C) and Greenhouse (D) ASVs.

# Legend of supplementary figures

[Supplementary Figure 1: Instruction manual for participating growers. Translated from Dutch. 3](#_Toc193450947)

[Supplementary Figure 2: Relative abundance of the 10 most abundant taxa in the strawberry flowers sampled during the Sabofleur project, based on a genus level. ‘Other taxa’ refers to the number of samples with a different genus. 4](#_Toc193450948)

[Supplementary Figure 3: Cumulative number of unique ASVs as a function of the number of strawberry flower samples obtained from four different cultivation types after ordering the samples from high to low abundances in every cultivation category. 4](#_Toc193450949)

[Supplementary Figure 4: Intra-farmer beta diversity. Similarly to between the cultivation types: variability is higher in covered cultivation systems. 5](#_Toc193450950)

[Supplementary Figure 5: Occupancy-occupancy plots for the different commercial cultivation types. A: Open fields vs greenhouses.B: Open fields vs Tunnels. C: Greenhouses vs Tunnels. Taxa occurring significantly more in one cultivation type based on Fisher’s test are depicted in dark blue. 8](#_Toc193450951)

[Supplementary Figure 6: Differential abundance for all taxa, significantly more prevalent in one cultivation type over another according to a pairwise Fischer test. 8](#_Toc193450952)

[Supplementary Figure 7: A: Log(Inverse Simpson Index) in function of surface area. The p-values are calculated using a glm-model after subsetting for cultivation type and organic vs conventional cultivation. B: Within farm beta diversity in function of the surface area of the strawberry field. The p-values were calculated using constrained analysis of principal coordinates (CAP), followed by ANOVA after subsetting for cultivation type and organic vs conventional cultivation. Linear trendlines and 95% confidence interval were added. 9](#_Toc193450953)

[Supplementary Figure 8: A) + B) Occupancy-occupancy plots for both pollinator exclusion experiments. Open field flowers vs honeybee foragers (A) and tunnel flowers vs pollinators (B). C) + D) Differential abundance for all taxa, significantly more prevalent in flowers or pollinators in the open field (C) or the tunnel (D), according to a pairwise Fischer test. 10](#_Toc193450954)

[Supplementary Figure 9: Relative abundance of Extraction kit blanks (NC_kit), PCR blanks (NC_pcr), and sequencing blanks (NC_seq). Below each bar, the total number of read counts for that respective blank sample is depicted. The other blanks were empty. 11](#_Toc193450955)

[Supplementary Figure 10: Elbow method from Shade et al (2019) (39), employed to determine the core taxa per cultivation type. Elbows were determined based on Bray-Curtis similarity on a visual basis for Wild (A), Open Air (B), Tunnel (C) and Greenhouse (D) ASVs. 12](#_Toc193450956)

# Legend of supplementary tables

[Supplementary Table 1: Core ASVs across all cultivation types, determined using the occupancy-abundance elbow approach developed by Shade et al. (2019) (39). If the ASV is a core ASV in the respective cultivation type, it is depicted with “✔”. 5](#_Toc193450957)
